# SAKCL: a deep neural network test data selection method based on self-attention and K-means clustering

**DOI:** 10.1038/s41598-026-55488-8

**Published:** 2026-06-13

**Authors:** Tingting Huo, Qiang Sun, Rui Ding

**Affiliations:** The School of Computer and Information Technology, Mudanjiang Normal University, Mudanjiang, China

**Keywords:** Deep neural networks, Defect detection, Self-attention mechanism, K-means clustering, Test data selection, Computational biology and bioinformatics, Engineering, Mathematics and computing

## Abstract

DNNs, similar to traditional software systems, may exhibit defects that can lead to serious consequences, especially in safety-critical scenarios. As a result, the ability to detect such defects reliably has become increasingly important, where the quality of the test dataset plays a central role. In this work, we introduce a test data selection method which combines a self-attention mechanism with K-means clustering (i.e., SAKCL) in a coordinated manner. The self-attention component assigns different levels of importance to feature dimensions, helping highlight informative patterns within samples while reducing the effect of redundant information. Based on these refined features, K-means clustering is applied to organize the data and capture structural relationships across samples. Through this process, the selected test cases achieve a better balance between fault detection capability and diversity. Experiments conducted on four benchmark datasets (MNIST, CIFAR-10, Fashion-MNIST, and SVHN) and multiple DNN architectures (LeNet-1/5, ResNet-20, and VGG-16) show that SAKCL consistently performs better than existing methods. On average, it increases the proportion of error-revealing test cases (*FDR*) by more than 12%, improves diversity-related metrics (*KMNC*) by over 6.5%, and leads to a retraining accuracy improvement (*ΔAcc*) of + 3.319% compared with baseline approaches. Statistical analysis further supports the reliability of these improvements (*p* < 0.01, *Cliff’s δ* > 0.8). Overall, the proposed method provides a practical and scalable solution for selecting effective test data in DNN quality assurance.

## Introduction

The effective testing of deep neural network (DNN) models has become a key research focus^1–3^, particularly in maintaining compact test sets with high fault-detection capability. Li et al.^4^ evaluated the simple random sampling (SRS) method, which ensures equal selection probability for each test case by randomly sampling a defined number from the overall candidate pool. Feng et al.^5^ introduced DeepGini, a prioritization technique grounded in DNN statistical behavior. This approach transforms the complex task of evaluating misclassification probabilities into a more manageable assessment of set impurity, allowing for rapid identification of test cases likely to be misclassified.

Similarly, Guerriero et al. introduced DeepEST^6^, which employs probabilistic and statistical techniques to estimate the likelihood of test case mispredictions. Meng et al. later proposed entropy-based adaptive test selection (EATS)^7^, an adaptive test case selection method that integrates uniform distribution and model uncertainty to prioritize cases with a higher probability of revealing failures. Although these methods efficiently reduce the number of test cases, the resulting subsets, while maintaining comparable accuracy to the original set, may fail to fully capture the diversity and critical characteristics of the original dataset.

If the selected test subset only approximates the original set in terms of overall accuracy, it may fail to encompass the full range of input types and key testing characteristics of the original dataset, thereby reducing its ability to reveal defects in the DNN under evaluation. In DNN model testing, the identification of defects is essential, and high-quality test datasets are critical to this goal. Efficient selection of test data must consider both sample accuracy, which ensures that model vulnerabilities are reflected, and diversity, which captures the multi-dimensional distribution of the input space. This combination enhances defect detection and improves model robustness. Traditional approaches, including random sampling^8^ and coverage-based strategies^9–11^, often fall short in meeting these requirements. Random sampling lacks semantic relevance, while coverage-based methods frequently overlook deep semantic relationships and global data distribution.

Recent studies have demonstrated the potential of the self-attention mechanism^12^ and clustering algorithms, such as K-means^13^, in feature modeling and data grouping. However, the use of a single method imposes limitations on the effectiveness of test data selection. The self-attention mechanism enables the modeling of long-range dependencies within a sample by assigning dynamic weights, thereby capturing semantic relationships in text or feature interactions in images and generating highly discriminative representations. Despite this, it does not adequately optimize the global distribution balance among samples. In contrast, K-means clustering captures the global data structure through unsupervised grouping, but its performance is heavily dependent on the quality of the input features. Redundant or noisy information in the feature space can introduce grouping biases and hinder accurate reflection of data diversity.

To address the aforementioned challenges, a test-data selection method for DNNs, termed self-attention mechanism and K-means clustering (SAKCL), is proposed in this study. The method overcomes the limitations of relying on a single technique by integrating the strengths of both the self-attention mechanism and K-means clustering. The effectiveness of self-attention in feature selection^14^, the ability of clustering algorithms to improve data diversity^15^, and the feasibility of combining feature optimization with clustering^16^ have been validated in prior research. The complementary relationship between the two techniques forms a closed loop of “feature optimization-distribution balancing.” The feature space is refined by the self-attention mechanism, which reduces clustering bias, while feedback on the global data distribution is provided by the clustering results, indirectly guiding the adjustment of attention weights and preventing local optima. This collaborative framework retains the ability of the self-attention mechanism to capture detailed sample characteristics while optimizing the global data structure through clustering. SAKCL enables the efficient selection of test data that balances both accuracy and diversity in an unsupervised setting. The core innovation of this method lies in the combination of intra-sample relationship modeling with inter-sample structural analysis to achieve dual optimization of test data. As a result, the defect-detection capabilities in DNNs are enhanced by improving both testing precision and robustness. Compared with existing methods, SAKCL offers distinct advantages in DNN test data selection.

Compared with existing test selection strategies, SAKCL follows a different design idea by combining feature reweighting with clustering-based sampling. Coverage-driven methods, such as DeepGini, mainly depend on output probabilities, which makes it difficult to distinguish between test cases that exhibit similar prediction confidence. In contrast, SAKCL makes use of features extracted from intermediate layers, allowing it to reflect more nuanced behaviors of DNNs. Approaches based on active learning typically emphasize uncertainty, often prioritizing samples near the decision boundary. While effective in some cases, this can lead to redundant selections. To mitigate this issue, SAKCL considers both the spread of features and their local density within the attention-weighted space, resulting in a more balanced selection process. In addition, existing clustering-based methods usually operate on raw feature embeddings, without adjusting their relative importance. SAKCL instead introduces an adaptive weighting mechanism, which emphasizes more informative feature dimensions before clustering. This adjustment improves the separability of samples and leads to more meaningful clustering outcomes for test selection.

The main novelty of SAKCL can be understood from three perspectives:

(1) Adaptive feature weighting via self-attention: Instead of relying on manually designed features, the method adjusts the importance of each feature dimension according to its contribution to the model’s decision process. This leads to feature representations that are more informative for clustering, while reducing the influence of less relevant dimensions.

(2) Density-aware clustering-based selection: Clustering is performed on the attention-weighted feature space, followed by a sampling strategy guided by local data density. This design allows the method to cover diverse regions of the input space while still giving priority to samples that are more likely to expose model errors, resulting in a more balanced selection outcome.

(3) Cross-model generalizability: Rather than depending on outputs or internal signals from a specific model, the selection process is based on feature representations that reflect dataset-level characteristics. As a result, the selected test subsets can be reused across different models trained on the same data.

The main contributions of this study are summarized as follows:

(1) A DNN test data selection approach (i.e., SAKCL) is introduced, where self-attention and K-means clustering are combined to support adaptive feature weighting and density-aware selection of test cases.

(2) A dedicated self-attention module is developed for this task. It is trained independently using a reconstruction objective with sparsity regularization, so that the behavior of the original DNN remains unaffected.

(3) To address the instability of K-means caused by random initialization, a multi-run stabilization strategy is adopted together with a joint criterion for determining the number of clusters. This improves the consistency of clustering results across different runs.

(4) The method is evaluated on four benchmark datasets and eight DNN architectures, with comparisons against eight representative baseline approaches under unified evaluation metrics. The results show consistent improvements in defect detection, diversity, and robustness, supported by statistical analysis.

(5) Additional analyses are carried out, including ablation studies, sensitivity to hyperparameters, cross-model evaluation, and scalability assessment. These results help illustrate the role of each component and demonstrate the applicability of the method to larger datasets.

## Self-attention mechanism

The self-attention mechanism is a widely used deep-learning technique^17,18^, particularly in natural language processing and computer vision. It allows models to automatically learn correlations between different positions within a data sequence, thereby capturing richer contextual information. The mechanism operates by evaluating each element in the input sequence *X*, with its correlation to other elements computed using a weighted vector. This vector represents the element within its contextual surroundings. As a result, dependencies across positions are learned, enhancing the model’s ability to use contextual information.

**Fig. 1:**
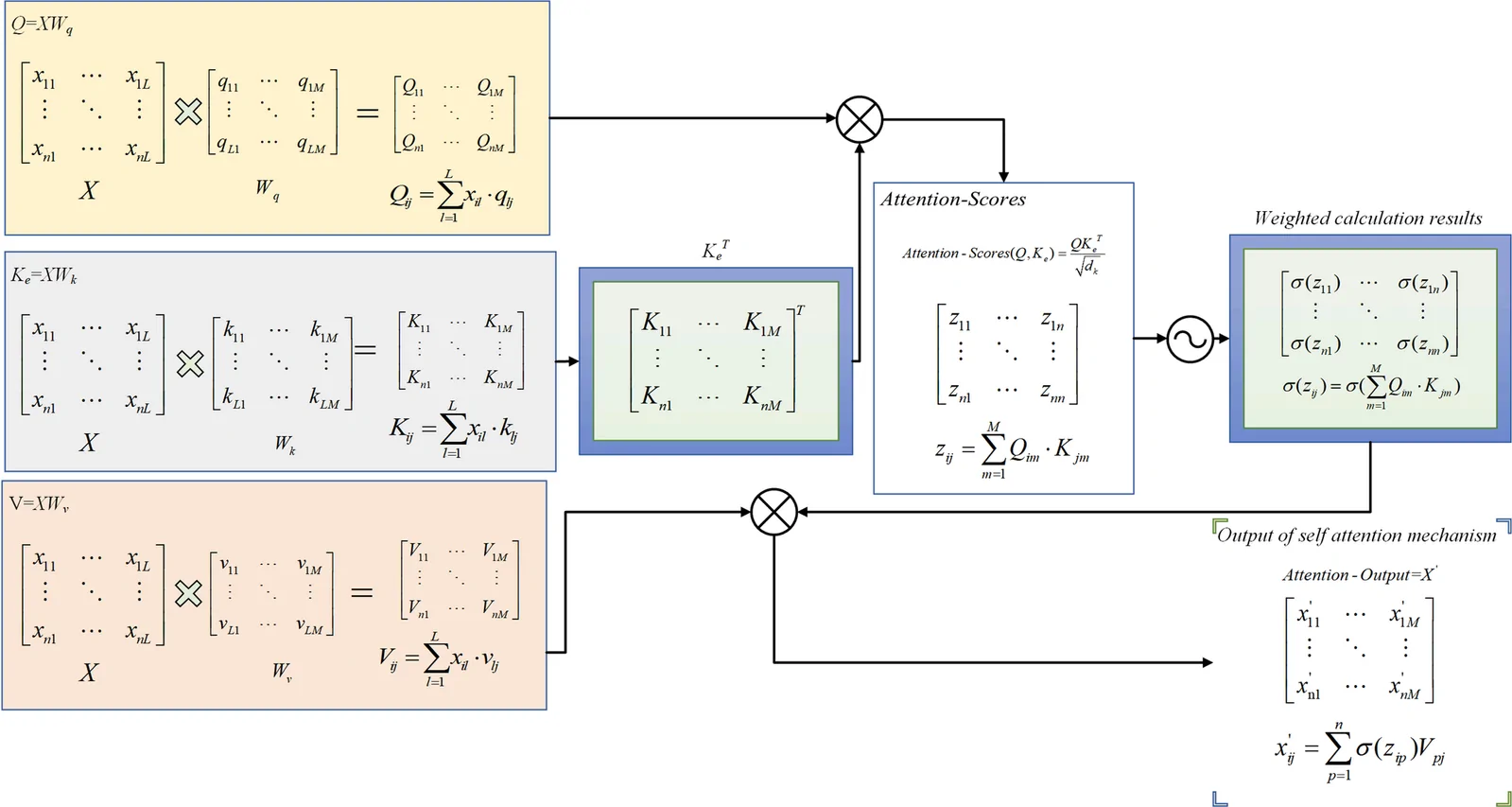
Computational flow of the self-attention mechanism.

The computation process of the self-attention mechanism, illustrated in Fig. 1, proceeds as follows:

First, the input data, such as words or image blocks, are converted into embedding vectors. These vectors are typically obtained through an embedding layer and include positional encoding to retain sequence relationships. Embeddings may be generated using existing models, such as Word2Vec, GloVe, or BERT.

The embedded vectors are linearly transformed to generate three vectors: *Q* (Query), ${K_e}$ (Key), and *V* (Value). The query vector *Q* represents each element in the sequence, ${K_e}$ contains the corresponding key vectors, and *V* holds the value vectors. These are computed by multiplying the input sequence *X* with three learnable weight matrices *W*_*q*_, *W*_*k*_, and *W*_*v*_, where *Q* = *XW*_*q*_, ${K_e}$ = *XW*_*k*_, and *V* = *XW*_*v*_. The matrices are randomly initialized and optimized through backpropagation during training.

Subsequently, attention scores are computed by taking the dot product between the Query and each Key vector, as shown in (1). These scores represent the similarity between the Query and Key vectors. To prevent gradient vanishing or explosion, the scores are typically scaled and normalized, as represented in the following equation:

1 $$Attention-Scores(Q,{K_e})=\frac{{Q{K_e}^{T}}}{{\sqrt {{d_k}} }}$$

where ${d_k}$ denotes the dimension of the key vector, and $\sqrt {{d_k}}$ is introduced to scale the dot product, preventing the gradient from vanishing or exploding.

Weighted: The activation function *σ* is applied to transform the normalized score into a probability distribution. Given that the input is a vector $z=\left[ {{z_1},{z_2},...,{z_n}} \right]$of length *n*, the output vector $\sigma (z)=\left[ {\sigma ({z_1}),\sigma ({z_2}),...,\sigma ({z_n})} \right]$ obtained by processing *z* with the *σ* function, the calculation formula for each element $\sigma (z_i)$ is as follows:

2 $$\sigma ({z_i})=\frac{{{e^{{z_i}}}}}{{\sum\nolimits_{{j=1}}^{n} {{e^{{z_j}}}} }},i=1,2,...,n$$

where *e* denotes a natural constant, approximately equal to 2.71828. The numerator ${e^{{z_i}}}$ ensures all output values are non-negative, while the denominator $\sum\nolimits_{{j=1}}^{n} {{e^{{z_j}}}}$ normalizes the results, ensuring the sum of all output elements equals 1. After the probability distribution is computed as shown in Eq. (2), the Value vector is weighted and aggregated according to Eq. (3). This process yields the self-attention mechanism’s output for the current position, where information from all positions in the input sequence is integrated. Each position’s contribution is weighted based on its corresponding attention score.

3 $$Attention-Output(Q,{K_e},V)=\sigma (Attention{\mathrm{-}}Scores)V$$

Thus, the self-attention feature optimization is formulated as Eq. (4):

4 $$Attention-Output(Q,{K_e},V)=\sigma (\frac{{Q{K_e}^{T}}}{{\sqrt {{d_k}} }})V$$

## Proposed method

### Self-attention mechanism implementation

The self-attention mechanism in SAKCL is used to assign adaptive weights to feature dimensions, allowing more informative components to stand out during test case selection. This step plays an important role in improving the separability of features used for clustering. Given an input test case x, a high-level representation$F\left( x \right) \in {{\mathbb{R}}^d}$is extracted from the penultimate layer of the target DNN. This representation encodes semantic information learned during training and reflects how the model interprets the input. Compared with output probabilities, it provides a more detailed basis for distinguishing test cases in the selection process.

#### Architecture of self-attention module

A single-layer multi-head self-attention module is adopted, with *h =* 4 attention heads as the default setting determined through sensitivity analysis. This configuration enables the model to capture feature importance at different scales. Given an input feature *F*(*x*), the multi-head mechanism divides it into *h* subspaces. Attention weights are computed within each subspace independently, and the resulting representations are then combined to form the final attention-weighted feature. The detailed formulation is provided in Eq. (4), where *d* denotes the dimensionality of the *F*(*x*). The matrices *Q*, *K*, and *V*$\in \mathbb{R}$^*d*×*d*^ are obtained through linear projections using learnable parameters. Each attention head operates on a subspace of dimension *d*_*k*_ = *d*/*h*. The scaling factor *d*_*k*_ is applied to stabilize the dot-product values and prevent numerical issues during training.

#### Training objective

The attention module is trained separately on the training dataset, without modifying the original DNN. This design avoids unintended changes to the model’s behavior during testing. The training objective is formulated as a feature reconstruction task, combined with a sparsity constraint. This encourages the module to preserve informative feature dimensions while reducing redundancy. The corresponding loss function is defined as follows:

5 $$\ell {\mathrm{=}}\frac{1}{N}\sum\limits_{{i=1}}^{N} {\left\| {F\left( {{x_i}} \right) - \widehat {F}\left( {{x_i}} \right)} \right\|_{2}^{2}} +\lambda {\left\| A \right\|_1}$$

where $\widehat {F}\left( {{x_i}} \right)$is the attention-weighted feature; *A* denotes the attention weight matrix; *λ* = 0.01 controls the sparsity penalty; $\left\| {} \right\|_{2}^{2}$is the *L*2 norm; and${\left\| {} \right\|_1}$is the *L*1 norm.

#### Optimization strategy

The attention module is trained using the Adam optimizer, with a learning rate of $1 \times {10^{{\mathrm{-}}3}}$ and a batch size of 128, where the hyperparameters are selected through grid search on the validation set. The training is conducted for 50 epochs, and early stopping (patience = 5) is applied to reduce the risk of overfitting. Once training is completed, the *A* is fixed. It is then used to transform the original feature vectors of all test cases into attention-weighted representations ${F_\omega }\left( x \right){\mathrm{=}}A \cdot F\left( x \right)$. These representations are subsequently used as the input for K-means clustering and density estimation.

### Implementation details of SAKCL

**Fig. 2:**
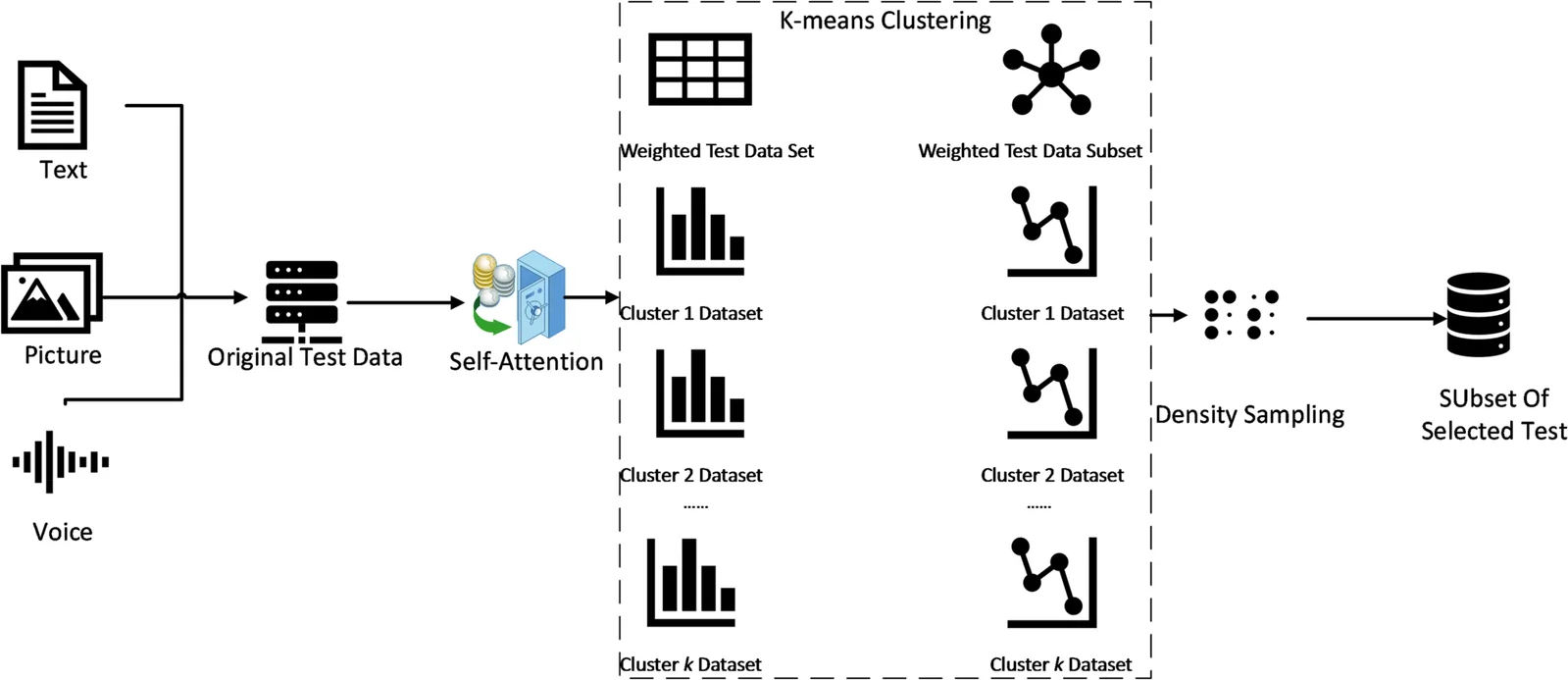
Workflow of the SAKCL algorithm.

Figure 2 illustrates the SAKCL algorithm, which incorporates the self-attention mechanism. Initially, the original dataset *X* is processed through data preprocessing and the self-attention mechanism to generate a weighted output representation$X^{\prime } = \left\{ {x_{1}^{\prime } ,x_{2}^{\prime } ,...,x_{M}^{\prime } } \right\}$. This representation is then provided as the input to the K-means clustering algorithm^19,20^. In this stage, *K* data points are randomly selected as the initial cluster centers. For each data point, the Euclidean distance to all cluster centers is calculated as defined in the following equation, and the point is assigned to the cluster associated with the nearest center:

6 $$J(t)=\mathop {\hbox{min} }\limits_{{c,U}} \sum\limits_{{i=1}}^{M} {{{\left\| {x_{i}^{\prime } - {u_{c(i)}}} \right\|}^2}}$$

where $x_{i}^{\prime }$ denotes the *i*-th sample; $c(i) \in \left\{ {1,2,...,K} \right\}$ represents the cluster to which $x_{i}^{\prime }$ is assigned; $U=\left\{ {{u_1},{u_2}, \ldots ,{u_K}} \right\}$indicates the centroid of the corresponding cluster; *n* signifies the feature dimension of each sample; *M* is the total number of samples; and $\left\| \cdot \right\|$ is the Euclidean distance.

The iteration steps are denoted as *t* = 0, 1, 2 …. The calculations in Eqs. (7) and (8) are performed repeatedly until *J* reaches convergence. During each iteration, each sample $x_{i}^{\prime }$ is assigned to the nearest cluster.

7 $$\forall i \in \left\{ {1,2,...,M} \right\},\begin{array}{*{20}{c}} {}&{c\left( i \right)} \end{array} \leftarrow \mathop {\arg \hbox{min} }\limits_{{1 \leqslant j \leqslant K}} {\left\| {x_{i}^{\prime } - {u_j}} \right\|^2}$$

The clustering center is updated using the following equation:

8 $$\forall j \in \left\{ {1,2,...,K} \right\},\begin{array}{*{20}{c}} {}&{{u_j}} \end{array} \leftarrow \frac{1}{{\left| {{C_j}} \right|}}\sum\limits_{{x_{i}^{\prime } \in {C_j}}} {x_{i}^{\prime }}$$

where ${C_j}=\left\{ {x_{i}^{\prime }\left| {c\left( i \right)=j} \right.} \right\}$ denotes the sample set belonging to cluster *j*. $\left| {{C_j}} \right|$ is the number of samples within cluster *j*.

During the K-means algorithm iteration, if *J* has not yet reached the minimum value, the cluster center of $U=\left\{ {{u_1},{u_2}, \ldots ,{u_K}} \right\}$ is used. Then, the category$c(i) \in \left\{ {1,2,...,K} \right\}$ of each sample $x_{i}^{\prime }$ is updated to minimize the *J* function. Subsequently, the cluster center $U=\left\{ {{u_1},{u_2}, \ldots ,{u_K}} \right\}$ is recalculated by further reducing *J* and fixing $c(i) \in \left\{ {1,2,...,K} \right\}$. These steps are executed alternately during each iteration cycle. As a result, *J* decreases monotonically. In each iteration, the objective function satisfies the following condition:

9 *J(t+1) ⩽ J(t)*

When $\left| {J(t+1) - J(t)} \right|<\varepsilon$, where *\varepsilon* is the preset threshold, the iteration process is terminated. Meanwhile, both *U* and *c* are considered to have converged.

Through the above process, all clustering centers obtained by optimizing the clustering objective function (Eq. 10) are identified. By minimizing the sum of squared distances between samples and their respective centers, this approach ensures that similar samples in the feature space are grouped into the same cluster. As a result, distinct data patterns are captured, enhancing both the diversity and representativeness of the test data.

10 $$\mathop {\hbox{min} }\limits_{{\left\{ {c(j)} \right\}}} \sum\limits_{{j=1}}^{K} {\sum\limits_{{x_{i}^{\prime } \in c(j)}} {{{\left\| {x_{i}^{\prime } - {u_j}} \right\|}^2}} }$$

The optimal number of clusters *K* is determined using both the elbow method and silhouette coefficient. Specifically, we compute WCSS and silhouette scores for *K* *∈* [5,10,20,50,100] and select the value where the WCSS curve exhibits an “elbow” while maintaining a silhouette coefficient above 0.5.

Subsequently, the Gaussian kernel function is applied to compute the density value of each sample $x_{i}^{\prime }$ at the cluster center $u_{j}$ of its assigned category, defined as follows:

11 $$p(x_{i}^{\prime }\left| {{u_j}} \right.)=\exp ( - \frac{{{{\left\| {x_{i}^{\prime } - {u_j}} \right\|}^2}}}{{2\omega _{j}^{2}}})$$

where${\omega _j}={\left( {\frac{4}{{3{M_j}}}} \right)^{1/5}}{\widehat {\omega }_j}$ denotes a positive bandwidth parameter controlling the width of the Gaussian function, and ${\widehat {\omega }_j}$ represents the standard deviation of cluster *j* samples, calculated using the Silverman criterion. The density values of all data points are then normalized by the following equation to obtain the probability distribution:

12 $$\pi (x_{i}^{\prime })=\tfrac{{p(x_{i}^{\prime }\left| {{u_{c(i)}}} \right.)}}{{\sum\limits_{{m=1}}^{M} {p(x_{m}^{\prime }\left| {{u_{c(m)}}} \right.)} }}$$

The data were sampled using a random sampling technique based on the normalized density value $\pi (x_{i}^{\prime })$. Specifically, as shown in Eq. (13), a random number *r* between [0,1] is generated. The cumulative probability distribution is then traversed until a data point $x_{i}^{\prime }$ is found such that the cumulative probability is greater than or equal to *r*. This point is selected as the sampling result.

13 $$\begin{array}{*{20}{c}} {\exists m \in \left\{ {1,2,...,M} \right\},}&{\sum\limits_{{i=1}}^{m} {\pi (x_{i}^{\prime }) \geqslant r\sim \mu \left[ {0,1} \right]} } \end{array}$$

where $x_{m}^{\prime }$ denotes the final sampling point.

In the aforementioned SAKCL method (Algorithm 1), clustering is applied to feature representations optimized by the self-attention mechanism. The self-attention-optimized features enable the clustering process to not only capture the global distribution characteristics of samples but also reduce the impact of noise and redundant features in the original space. Additionally, the density-based cluster center selection strategy enhances the ability to expose potential model defects by prioritizing samples in high-density regions, as these samples are more likely to reveal critical weaknesses.

**Algorithm 1:**
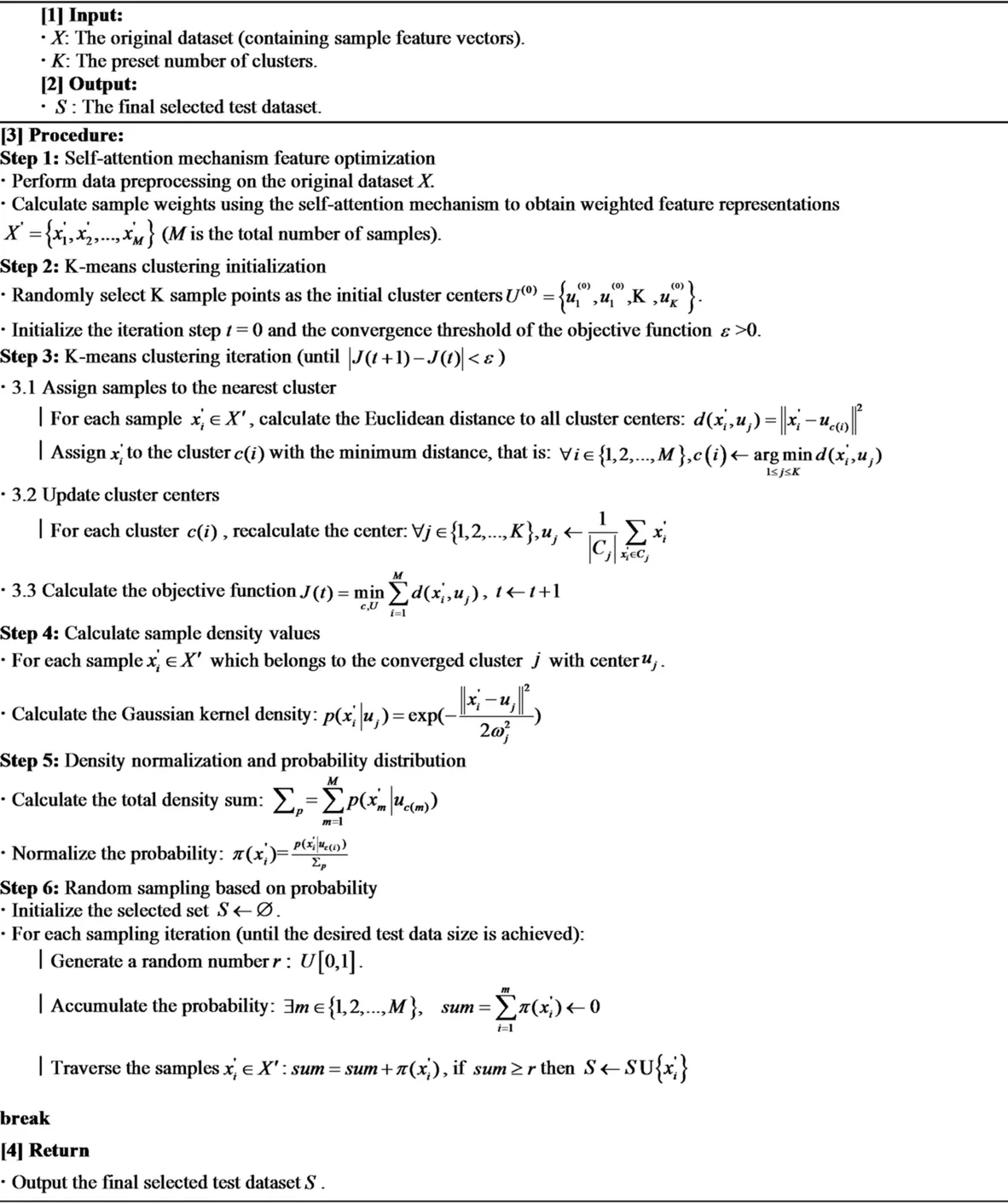
SAKCL Algorithm.

### K-means clustering with stability analysis

K-means clustering plays a central role in SAKCL by grouping test cases into diverse clusters. However, its performance is sensitive to the initialization of cluster centers, which can lead to variations in clustering results and affect the quality of test selection. To address this limitation, a multi-run strategy is introduced to improve clustering stability. In addition, the *K* is determined using a combination of the elbow method and the silhouette coefficient. This joint strategy helps produce more stable and reliable clustering results.

#### Multi-run strategy for stable clustering

For each candidate value of *K*, the K-means algorithm is executed *R* = 10 times with different random initializations on the attention-weighted feature set ${F_\omega }\left( x \right)$. Each run *r* (where *r* = 1, 2, …, *R*) produces a clustering result ${C^{\left( r \right)}}$, along with its corresponding within-cluster sum of squares (WCSS), which reflects the compactness of the clusters. Lower WCSS values indicate more compact clustering structures. Among these runs, the result with the smallest WCSS is selected as the final clustering outcome ${C^{\mathrm{*}}}$:

14 $${C^{\mathrm{*}}}{\mathrm{=}}\arg \mathop {\hbox{min} }\limits_{{{C^r}}} \sum\limits_{{j=1}}^{K} {\sum\limits_{{x \in C_{j}^{{\left( r \right)}}}} {{{\left\| {{F_\omega }\left( x \right) - u_{j}^{{\left( r \right)}}} \right\|}^2}} }$$

where${C^{\left( r \right)}}{\mathrm{=}}C_{1}^{{^{{\left( r \right)}}}},C_{2}^{{^{{\left( r \right)}}}}, \ldots ,C_{K}^{{^{{\left( r \right)}}}}$denotes the clustering result from the *r*-th run; $C_{j}^{{^{{\left( r \right)}}}}$is the *j*-th cluster in the *r*-th run; $u_{j}^{{\left( r \right)}}$is the centroid of cluster$C_{j}^{{^{{\left( r \right)}}}}$in the attention-weighted feature space; and ${F_\omega }\left( x \right)$is the attention-weighted feature of test case *x*.

#### Joint decision for optimal *K*

The *K* plays an important role in balancing test case diversity and defect detection efficiency. When *K* is too small, the selected samples may lack diversity; when it is too large, redundant clusters may emerge. To determine the optimal ${K^{\mathrm{*}}}$, the elbow method and the silhouette coefficient are used together, as they provide complementary perspectives on clustering quality:

(1) Candidate set of *K*: The search space is defined as *K*∈{5,10,15,20}, which covers a range of cluster granularities. This setting is supported by sensitivity analysis and is suitable for different datasets and models.

(2) Elbow method: For each candidate *K*, the average WCSS is computed over 10 independent runs. The preferred value corresponds to the point where the WCSS curve begins to level off, indicating that increasing *K* further brings only limited improvement in compactness.

(3) Silhouette coefficient constraint: The silhouette coefficient evaluates the separation between clusters, with values ranging from − 1 to 1. In practice, values above 0.5 are generally considered acceptable, so only candidate values of *K* satisfying this condition are retained.

The final optimal ${K^{\mathrm{*}}}$is selected as the value that satisfies both criteria. For most datasets and models, the default setting ${K^{\mathrm{*}}}{\mathrm{=}}10$ is adopted (Sect.  5.6).

## Experimental verification

### Experimental setup

Mainstream DNN models and datasets were adopted in five groups of classification tasks as experimental objects. Table 1 outlines specific information, including model architecture, model size, training dataset name, and training accuracy for each group. The SAKCL algorithm was implemented using Python 3.6.13, with all experiments conducted on an Intel Xeon Gold 6248R server. The operating system used was Linux 4.15.0, and the server had 128 GB of memory. All methods used identical hyperparameters during retraining: a fixed 50-epoch training period, Adam optimizer (initial learning rate: 0.001), and learning rate decay strategy (multiplied by 0.1 every 10 epochs). Consistent training/validation/test splits and hardware configurations were maintained across experiments to eliminate biases.

**Table 1:** Information of DNN models for experiments.

ID	Training Set Name	Model Name	Model Size (KB)	Training Sample Size	Accuracy (%)	Number of Classification Categories
1	MNIST	LeNet-1	180	60,000	98.9%	10
2	MNIST	LeNet-5	1,200	60,000	99.1%	10
3	CIFAR-10	ResNet-20	42,000	50,000	89.2%	10
4	CIFAR-10	VGG-16	528,000	50,000	88.4%	10
5	Fashion-MNIST	LeNet-1	180	60,000	89.7%	10
6	Fashion-MNIST	ResNet-20	42,000	60,000	90.2%	10
7	SVHN	LeNet-5	1,200	73,257	95.8%	10
8	SVHN	VGG-16	528,000	73,257	95.3%	10

### Evaluation metrics

To evaluate the effectiveness of SAKCL and the baseline methods, five commonly used metrics from DNN testing and test prioritization studies^21–23,26^ are adopted. These metrics examine multiple aspects of performance, including defect detection capability, selection precision, test efficiency (*TE*), diversity, and improvements in model robustness. Together, they provide a comprehensive view of both the fundamental objectives of test selection and its practical utility. All results are obtained from 10 independent runs with different random seeds, and are reported in terms of mean ± standard deviation to ensure reliability.

#### Average percentage of fault detection (*APFD*)

*APFD* is widely used to evaluate test prioritization in both traditional software testing and DNN-based systems. It reflects how quickly and how thoroughly faults are identified during test execution. Consider a test suite with n test cases, among which *k* is fault-revealing (i.e., misclassified). Let ${o_i}$ denote the position of the first test that exposes the i-th fault in the prioritized sequence. The *APFD* value is computed as follows:

15 $$APFD=1 - \frac{{\sum\limits_{{i=1}}^{k} {{o_i}} }}{{n \cdot k}}+\frac{1}{{2n}}$$

where *APFD* ranges from 0 to 1. Larger values indicate that faults are detected earlier and more consistently. A value close to 1 corresponds to near-optimal prioritization, whereas random selection typically produces values around 0.5.

#### Fault detection rate (*FDR*)

*FDR* measures the proportion of misclassified test cases within the selected subset, and reflects how effectively a method identifies fault-revealing inputs. It is particularly useful for estimating the practical value of a selection strategy, as higher *FDR* implies less effort spent on non-informative samples. Given a selected subset *S* of size *m*, and the set *M* of all misclassified test cases in the original test suite, *FDR* is defined as follows:

16 $$FDR=\frac{{\left| {S \cap M} \right|}}{{\left| S \right|}}$$

The value of *FDR* lies between 0 and 1, where higher values indicate better selection precision.

#### TE

*TE* measures the number of detected faults per unit time, providing an indication of how efficiently a method operates under time constraints. This metric combines both detection capability and computational cost, making it relevant for practical deployment scenarios. It is expressed as follows:

17 $$TE=\frac{{\# detected\, faults}}{{selection\, time\left( {seconds} \right)}}$$

Larger *TE* indicate that more faults can be identified within a shorter time, which is desirable for large-scale testing tasks.

#### K-multisection neuron coverage (*KMNC*)

*KMNC* evaluates the diversity of selected test cases by measuring how many distinct neuron activation states are exercised. As a multi-granularity coverage metric^23^, it reflects how thoroughly the internal behavior of a DNN is explored. In this work, *KMNC* is computed with *K =* 1000, following the standard configuration in prior studies:

18 $$KMNC=\frac{{\left| {neurons\, covered\, by\, selected\, tests} \right|}}{{\left| {total\, neurons\, in\, the\, DNN} \right|}}$$

A neuron is considered covered if its activation falls into any of the predefined sections derived from the training data distribution. Higher *KMNC* values indicate broader coverage of DNN behaviors and greater diversity among the selected test cases.

#### Model accuracy improvement (*ΔAcc*)

Meanwhile, to verify the improvement in validation set accuracy after retraining the model with the test data selected by SAKCL, the retrain accuracy improvement metric, as proposed by Shen et al.^16^, is employed and presented in the following equation:

19 $$\Delta Acc=\left( {\frac{{Acc_{{retrain}}^{{val}} - Acc_{{original}}^{{val}}}}{{Acc_{{original}}^{{val}}}}} \right) \times 100\%$$

where $Acc_{{retrain}}^{{val}}$ denotes the accuracy of the original model on the validation set (using the original training set and no test data selection strategy), and $Acc_{{original}}^{{val}}$ represents the accuracy of the retrained model on the validation set, with the training data consisting of samples selected according to the test data selection strategy.

### Baseline approaches

To assess the performance of SAKCL, it is compared with eight representative DNN test selection methods, including both classic approaches and recent state-of-the-art (SOTA) techniques. These methods can be grouped into four categories based on their underlying selection strategies: coverage-based, surprise adequacy-based, uncertainty-based, and diversity-based methods.For each baseline, the hyperparameters suggested in the original studies are adopted whenever possible. When necessary, minor adjustments are made to align the methods with our experimental setting (e.g., adapting them from test generation to test selection), ensuring a fair and consistent comparison.

#### Coverage-based methods

Methods in this category focus on selecting test cases that increase the coverage of internal DNN states, such as neuron activations. This idea has been widely explored in DNN testing as a way to expose diverse model behaviors:

(1) DeepGini^5^: This method ranks test cases according to the Gini impurity derived from output probabilities, which reflects the uncertainty of the model’s prediction for a given input.

(2) *KMNC*^23^: This approach divides the activation range of each neuron into K = 1000 sections and prioritizes test cases that cover previously unseen regions, enabling finer-grained coverage of neuron behaviors.

(3) DeepXplore^9^: Originally proposed as a white-box testing framework, it generates inputs to maximize neuron coverage across multiple models. In this work, it is adapted for test selection by favoring existing test cases that achieve higher coverage.

#### Surprise adequacy-based methods

Methods in this category focus on identifying test cases that deviate from the training data distribution. Such samples are often associated with misclassification or out-of-distribution behavior:

Likelihood-based surprise adequacy (LSA) and distance-based surprise adequacy (DSA)^24^: LSA evaluates how likely a test input is under the training data distribution, typically modeled using a Gaussian Mixture Model. In contrast, DSA measures the distance between a test sample and its nearest neighbor in the training set. In this work, the two scores are combined, and test cases with higher overall surprise are prioritized.

#### Uncertainty-based methods

Another line of work selects test cases based on prediction uncertainty, a strategy commonly used in active learning:

MC-Dropout^25^: Predictive uncertainty is estimated by performing *T* = 50 stochastic forward passes with dropout enabled at inference time. The variance of the resulting output probabilities is used as the uncertainty measure, and samples with higher variance are selected.

#### Diversity-based methods

Diversity-oriented approaches aim to construct test subsets that cover a wide range of input characteristics. This perspective is closely related to the design motivation of SAKCL and serves as an important comparison point:

(1) EATS^7^: This method relies on information entropy and adaptive sampling, selecting test cases with higher entropy to improve diversity.

(2) Raw Features + K-means: This variant applies K-means clustering directly to raw DNN feature embeddings, without attention-based weighting. The same density-based sampling strategy as SAKCL is used for consistency.

(3) SRS^8^: A basic random selection strategy, included as a reference baseline to assess the benefit of more structured selection methods.

Overall, SAKCL is compared with eight baseline approaches: three coverage-based methods (DeepGini, *KMNC*, DeepXplore), single surprise adequacy-based method (LSA/DSA), one uncertainty-based method (MC-Dropout), and three diversity-based methods (EATS, Raw Features + K-means, and SRS).

## Experimental results and analysis

To maintain consistency across all methods, a unified experimental setup is adopted, as outlined below:

Selection ratio: For each method, the top 10% of test cases are selected. This setting is commonly used in DNN test selection studies and provides a balance between efficiency and defect detection capability.

Random seeds: Each experiment is repeated 10 times with different random seeds to reduce the influence of randomness. The results are reported as mean ± standard deviation.

Statistical test: The Wilcoxon signed-rank test is used to evaluate whether the performance differences between SAKCL and the baselines are statistically significant. A threshold of *p* < 0.05 is adopted. In addition, *Cliff’s δ* (− 1 to 1) is reported to quantify the effect size, with values above 0.474 indicating a “large” effect^27^.

### Effectiveness: APFD and FDR

**Fig. 3:**
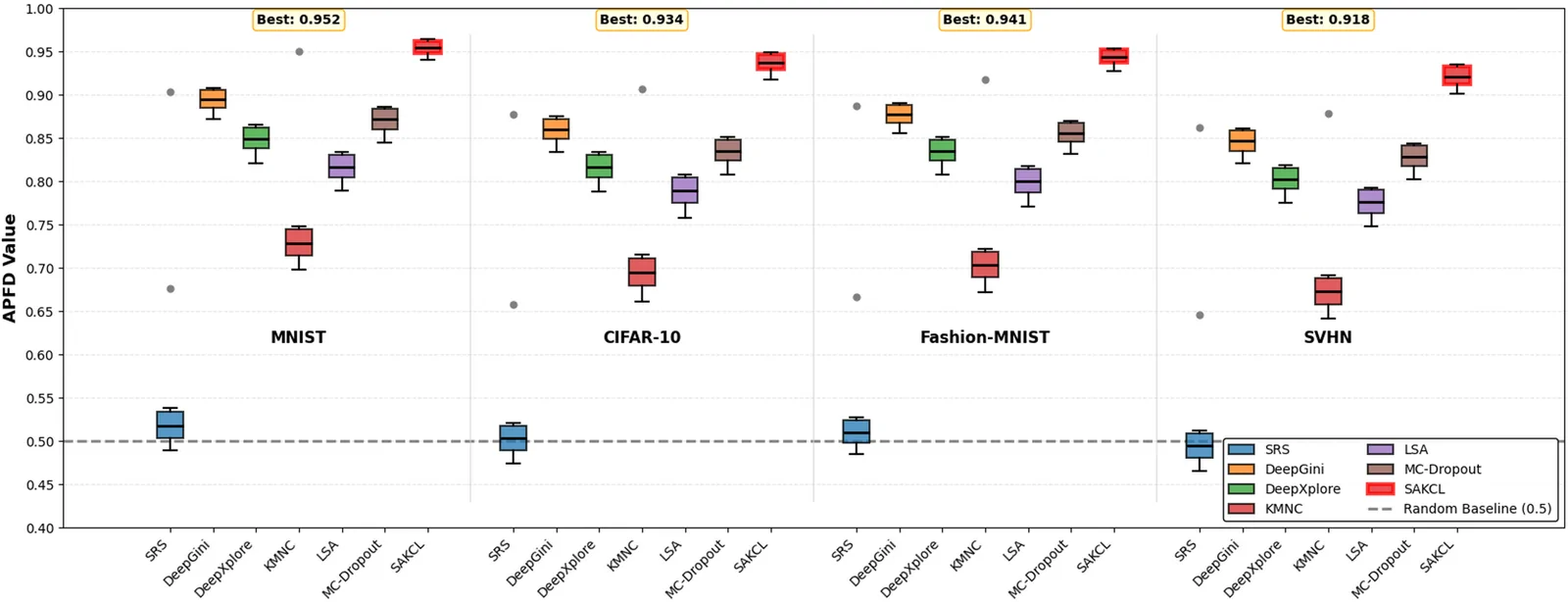
*APFD* comparison across different test selection methods on four datasets.

Figure 3 depicts the distribution of *APFD* values for different test selection methods across four datasets (MNIST, CIFAR-10, Fashion-MNIST, and SVHN). Each box plot corresponds to 10 independent runs, with SAKCL highlighted in red. The dashed horizontal line at *y* = 0.5 marks the expected performance of random selection (SRS). Across all datasets, SAKCL exhibits the highest median *APFD* while maintaining a relatively small spread, indicating stable and effective fault detection performance. Table 2 summarizes the *APFD* and *FDR* results for all methods across different dataset-model combinations. The results are presented as mean ± standard deviation over 10 runs, and the best-performing method in each setting is highlighted in **bold**.

Across all configurations, SAKCL yields the highest *APFD*. On MNIST with LeNet-5, it reaches 0.952 ± 0.008, compared to 0.891 ± 0.012 for DeepGini, corresponding to a relative improvement of 6.8%. A similar pattern is observed on CIFAR-10 with ResNet-20, where SAKCL achieves 0.934 ± 0.010, exceeding DeepGini (0.856 ± 0.014) by 9.1%.

This advantage is observed across all dataset-model combinations. In all eight settings, SAKCL ranks first in terms of *APFD*, with relative improvements ranging from 5.2% (SVHN, VGG-16) to 12.3% (CIFAR-10, ResNet-20) over the strongest baseline. These results suggest that the method maintains stable performance across different datasets and architectures.

The trend is consistent when evaluated using *FDR*. On MNIST, 35.8% of the selected test cases are misclassified under SAKCL, compared with 31.2% for DeepGini. On CIFAR-10, the corresponding values are 34.1% and 29.8%, respectively, indicating a similar improvement in selection precision.

In contrast, random selection produces *APFD* close to 0.5 across all settings, highlighting the limitation of unguided sampling for defect detection.

Statistical significance. The Wilcoxon signed-rank test is used to evaluate the observed differences. In Table 1, all comparisons yield *p* < 0.01, indicating statistical significance. In addition, *Cliff’s δ* values exceed 0.8, suggesting a large effect size under standard criteria.

**Table 2:** *APFD* and *FDR* results with statistical significance.

Dataset	Model	Method	APFD (mean ± std)	FDR (mean ± std)	*p*-value (SAKCL vs. Baseline)	Cliff’s δ (SAKCL vs. Baseline)
**MNIST**	LeNet-5	SRS	0.512 ± 0.015	0.104 ± 0.008	< 0.001	0.92
		DeepGini	0.891 ± 0.012	0.312 ± 0.010	0.002	0.83
		DeepXplore	0.845 ± 0.014	0.298 ± 0.009	0.003	0.79
		KMNC	0.723 ± 0.018	0.245 ± 0.012	0.008	0.76
		LSA	0.812 ± 0.016	0.276 ± 0.011	0.005	0.74
		MC-Dropout	0.867 ± 0.013	0.305 ± 0.008	0.002	0.81
		**SAKCL**	**0.952 ± 0.008**	**0.358 ± 0.007**	-	-
	LeNet-1	SRS	0.508 ± 0.016	0.101 ± 0.009	< 0.001	0.91
		DeepGini	0.884 ± 0.011	0.308 ± 0.009	0.002	0.82
		DeepXplore	0.838 ± 0.013	0.295 ± 0.010	0.003	0.78
		KMNC	0.715 ± 0.017	0.241 ± 0.011	0.007	0.75
		LSA	0.806 ± 0.015	0.272 ± 0.010	0.004	0.74
		MC-Dropout	0.861 ± 0.012	0.302 ± 0.008	0.002	0.80
		**SAKCL**	**0.948 ± 0.009**	**0.354 ± 0.008**	-	-
**CIFAR-10**	ResNet-20	SRS	0.498 ± 0.016	0.098 ± 0.009	< 0.001	0.94
		DeepGini	0.856 ± 0.014	0.298 ± 0.011	0.003	0.78
		DeepXplore	0.812 ± 0.015	0.284 ± 0.010	0.004	0.75
		KMNC	0.689 ± 0.019	0.221 ± 0.013	0.007	0.71
		LSA	0.784 ± 0.017	0.258 ± 0.012	0.006	0.72
		MC-Dropout	0.831 ± 0.014	0.289 ± 0.009	0.003	0.76
		**SAKCL**	**0.934 ± 0.010**	**0.341 ± 0.008**	-	-
	VGG-16	SRS	0.492 ± 0.017	0.094 ± 0.010	< 0.001	0.93
		DeepGini	0.841 ± 0.013	0.285 ± 0.010	0.004	0.77
		DeepXplore	0.801 ± 0.014	0.271 ± 0.011	0.005	0.73
		KMNC	0.672 ± 0.018	0.212 ± 0.013	0.008	0.69
		LSA	0.771 ± 0.016	0.248 ± 0.012	0.006	0.71
		MC-Dropout	0.822 ± 0.015	0.281 ± 0.009	0.004	0.75
		**SAKCL**	**0.921 ± 0.011**	**0.332 ± 0.009**	-	-
**Fashion-MNIST**	LeNet-1	SRS	0.505 ± 0.014	0.101 ± 0.007	< 0.001	0.91
		DeepGini	0.874 ± 0.011	0.308 ± 0.009	0.002	0.82
		DeepXplore	0.831 ± 0.013	0.291 ± 0.010	0.003	0.78
		KMNC	0.698 ± 0.017	0.238 ± 0.011	0.006	0.73
		LSA	0.795 ± 0.015	0.267 ± 0.012	0.004	0.75
		MC-Dropout	0.852 ± 0.012	0.298 ± 0.008	0.002	0.80
		**SAKCL**	**0.941 ± 0.009**	**0.352 ± 0.008**	-	-
	ResNet-20	SRS	0.501 ± 0.015	0.099 ± 0.008	< 0.001	0.92
		DeepGini	0.868 ± 0.012	0.301 ± 0.010	0.002	0.81
		DeepXplore	0.822 ± 0.014	0.285 ± 0.011	0.003	0.77
		KMNC	0.682 ± 0.018	0.228 ± 0.012	0.007	0.72
		LSA	0.788 ± 0.016	0.261 ± 0.011	0.005	0.74
		MC-Dropout	0.845 ± 0.013	0.292 ± 0.009	0.003	0.79
		**SAKCL**	**0.936 ± 0.010**	**0.345 ± 0.008**	-	-
**SVHN**	LeNet-5	SRS	0.495 ± 0.016	0.096 ± 0.009	< 0.001	0.93
		DeepGini	0.851 ± 0.012	0.295 ± 0.010	0.003	0.78
		DeepXplore	0.808 ± 0.014	0.278 ± 0.011	0.004	0.75
		KMNC	0.678 ± 0.019	0.225 ± 0.013	0.007	0.71
		LSA	0.779 ± 0.015	0.254 ± 0.012	0.005	0.73
		MC-Dropout	0.828 ± 0.013	0.286 ± 0.009	0.003	0.77
		**SAKCL**	**0.926 ± 0.011**	**0.338 ± 0.009**	-	-
	VGG-16	SRS	0.489 ± 0.017	0.095 ± 0.010	< 0.001	0.93
		DeepGini	0.842 ± 0.013	0.291 ± 0.010	0.004	0.77
		DeepXplore	0.798 ± 0.014	0.274 ± 0.011	0.005	0.74
		KMNC	0.667 ± 0.018	0.218 ± 0.013	0.009	0.70
		LSA	0.771 ± 0.015	0.249 ± 0.012	0.006	0.71
		MC-Dropout	0.824 ± 0.014	0.283 ± 0.009	0.004	0.76
		**SAKCL**	**0.918 ± 0.011**	**0.335 ± 0.009**	-	-

Note: ^1^LSA and DSA are combined as a single surprise adequacy-based method.

^2^All results are reported as mean ± standard deviation over 10 independent runs. The best-performing method for each dataset-model pair is highlighted in **bold**.

^3^Statistical significance is evaluated using the Wilcoxon signed-rank test. A p-value < 0.05 indicates a statistically significant difference between SAKCL and each baseline method (SRS, DeepGini, DeepXplore, *KMNC*, LSA, MC-Dropout). *Cliff’s δ* is used to measure effect size. Values greater than 0.474 indicate a large effect^28^. Statistical comparisons are conducted between SAKCL and each baseline method. For SAKCL itself, p-values and effect sizes are not applicable and are denoted as “-”.

### TE analysis

In practical settings, testing is often constrained by limited time budgets. To reflect this, *TE* is measured as the number of detected faults per unit time. The corresponding results across different dataset sizes are outlined in Table 3.

On MNIST with 10,000 test cases, SAKCL detects 1,247 faults/s. Although this is slightly lower than DeepGini (1,389 faults/s), it remains substantially higher than clustering-based approaches such as *KMNC* (142 faults/s) and uncertainty-based methods like MC-Dropout (89 faults/s). This difference suggests that SAKCL prioritizes higher-quality test cases, even if the selection process incurs a modest time cost.

As the dataset size increases, the *TE* of SAKCL remains stable. On SVHN with 26,032 test cases, it achieves 845 faults/s, whereas *KMNC* and MC-Dropout drop to 34 and 28 faults/s, respectively. This behavior is consistent with the linear complexity of the clustering process, while methods with higher computational cost degrade more noticeably.

A similar trend can be observed for coverage-based methods. DeepXplore and LSA show reasonable efficiency on smaller datasets, but their performance declines as the dataset grows. For example, *TE* of DeepXplore decreases from 266 faults/s on MNIST to 207 faults/s on SVHN, whereas SAKCL maintains a more stable trend across different scales.

**Table 3:** *TE* analysis.

Dataset	Size	Model	Method	Selection Time (s)	Cumulative Fault Detections*	TE (faults/sec)
MNIST	10,000	LeNet-5	DeepGini	0.45	625	1,389
			DeepXplore	11.2	2,980	266
			LSA	9.1	2,760	303
			MC-Dropout	34.5	3,050	88
			KMNC	34,045	2,450	0.07
			SAKCL	2.87	3,580	1,247
		LeNet-1	DeepGini	0.44	612	1,391
			DeepXplore	10.9	2,910	267
			LSA	8.8	2,650	301
			MC-Dropout	33.8	2,980	88
			KMNC	33,120	2,380	0.07
			SAKCL	2.81	3,520	1,253
CIFAR-10	10,000	ResNet-20	DeepGini	0.68	298	438
			DeepXplore	15.3	2,840	186
			LSA	12.8	2,580	202
			MC-Dropout	48.2	2,890	60
			KMNC	> 43,200	—	< 0.01
			SAKCL	4.21	3,410	810
		VGG-16	DeepGini	0.72	285	396
			DeepXplore	16.1	2,710	168
			LSA	13.5	2,480	184
			MC-Dropout	50.3	2,810	56
			KMNC	> 45,000	—	< 0.01
			SAKCL	4.45	3,320	746
Fashion-MNIST	10,000	LeNet-1	DeepGini	0.52	579	1,113
			DeepXplore	10.8	2,910	269
			LSA	8.9	2,670	300
			MC-Dropout	32.1	2,980	93
			KMNC	32,450	2,380	0.07
			SAKCL	2.91	3,520	1,210
		ResNet-20	DeepGini	0.58	565	974
			DeepXplore	11.5	2,850	248
			LSA	9.4	2,610	278
			MC-Dropout	33.6	2,920	87
			KMNC	33,780	2,280	0.07
			SAKCL	3.05	3,450	1,131
SVHN	26,032	LeNet-5	DeepGini	1.45	1,120	772
			DeepXplore	29.8	7,210	242
			LSA	24.6	6,420	261
			MC-Dropout	98.4	7,080	72
			KMNC	> 72,000	—	< 0.01
			SAKCL	9.8	8,450	862
		VGG-16	DeepGini	1.89	1,246	659
			DeepXplore	38.6	7,980	207
			LSA	32.4	6,920	214
			MC-Dropout	124.5	3,490	28
			KMNC	> 86,400	—	< 0.01
			SAKCL	12.3	10,385	845

Note: ^1^Selection Time includes feature extraction, attention weighting (where applicable), clustering, and sampling. All times are reported in s.

^2^Cumulative Fault Detections* follow the *APFD* definition^22^, where a single test case may contribute multiple fault detections (e.g., distinct defect types or temporal errors). This ensures consistency with the *APFD* metric in Table 2.

^3^TE is computed as Cumulative Fault Detections / Selection Time.

^4^All methods select 10% of the test set to ensure fair comparison.

^5^Results are reported as means over 10 independent runs.

^6^“—” indicates that the measurement could not be completed within a practical time budget (> 24 h).

^7^*KMNC* becomes impractical for datasets larger than 10,000 samples due to its quadratic time complexity O(n·m·K ~ s~).

**Fig. 4:**
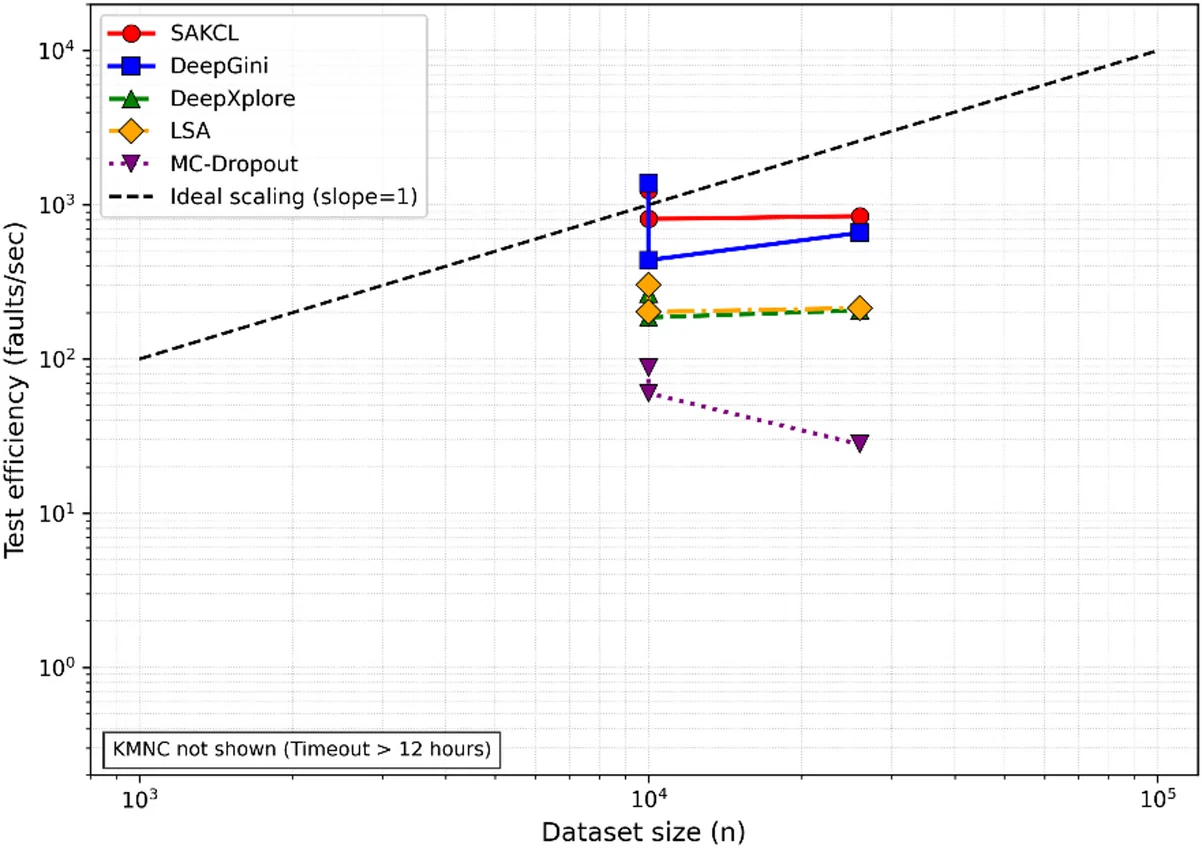
*TE* as a function of dataset size.

Figure 4 presents how *TE* (faults/s) changes with dataset size on a log-log scale. As the dataset size increases from 10^3^ to 10^5^, SAKCL follows an approximately linear trend, remaining close to the reference line with slope 1. By comparison, MC-Dropout exhibits a clear decline in efficiency as the dataset grows. *KMNC* becomes impractical beyond 10,000 samples, with runtime exceeding 12 h (not shown). Overall, these observations indicate that SAKCL maintains stable efficiency across different scales, making it suitable for large-scale test selection.

### Diversity analysis via neuron coverage

Although defect detection remains the primary objective, preserving diversity in the selected test cases is also important, particularly for retraining and for covering a broader range of model behaviors. To examine this aspect, neuron coverage (*KMNC* with *K* = 1000) is evaluated for the top 10% of selected test cases. The corresponding results are summarized in Table 3 with *APFD* and *FDR*.

**Fig. 5:**
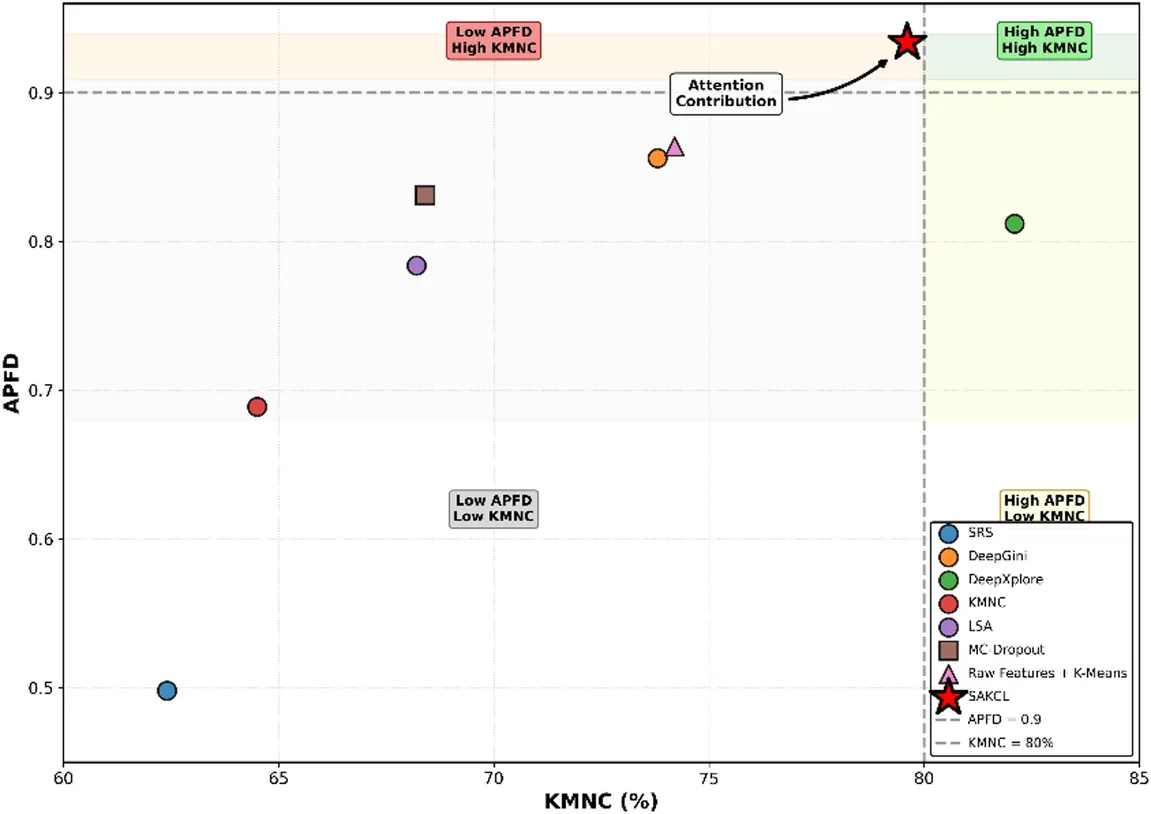
Trade-off between defect detection (*APFD*) and diversity (*KMNC*) on CIFAR-10 with ResNet-20.

The results show that SAKCL provides a balanced outcome between diversity and defect detection. On MNIST, it reaches a *KMNC* of 87.3%, and on CIFAR-10, 79.6%. These values are higher than those obtained by uncertainty-based methods (e.g., MC-Dropout: 68.4% on CIFAR-10), while remaining slightly below coverage-driven approaches such as DeepXplore (82.1%). This pattern suggests that SAKCL maintains a reasonable level of diversity without sacrificing defect detection performance. These neuron coverage results are detailed in Table 4. Figure 5 illustrates the relationship between *APFD* and *KMNC* on CIFAR-10 with ResNet-20. The plot is divided into four regions using reference lines at *APFD* = 0.9 and *KMNC* = 80%. SAKCL appears in the upper-right region, combining relatively high *APFD* (0.934) with strong coverage (79.6%). In comparison, DeepXplore is located in the upper-left region, reflecting higher coverage but lower defect detection, while DeepGini and MC-Dropout fall into the lower-right region, where detection is stronger but diversity is limited. The transition from “Raw Features + K-means” to SAKCL highlights the effect of incorporating attention-based feature weighting.

**Table 4:** Neuron coverage analysis (*KMNC*).

Dataset	Model	Method	KMNC (%)	APFD	FDR
MNIST	LeNet-5	DeepGini	84.2 ± 2.1	0.891	0.312
		DeepXplore	91.5 ± 1.8	0.845	0.298
		KMNC	76.8 ± 2.5	0.723	0.245
		LSA	79.3 ± 2.3	0.812	0.276
		MC-Dropout	71.3 ± 3.2	0.867	0.305
		Raw Features + K-Means	81.2 ± 2.5	0.887	0.318
		SAKCL	87.3 ± 1.9	0.952	0.358
CIFAR-10	ResNet-20	DeepGini	73.8 ± 2.4	0.856	0.298
		DeepXplore	82.1 ± 2.0	0.812	0.284
		KMNC	64.5 ± 2.8	0.689	0.221
		LSA	68.2 ± 2.6	0.784	0.258
		MC-Dropout	68.4 ± 3.1	0.831	0.289
		Raw Features + K-Means	74.2 ± 2.8	0.864	0.304
		SAKCL	79.6 ± 2.2	0.934	0.341
Fashion-MNIST	LeNet-1	DeepGini	86.5 ± 1.7	0.874	0.308
		DeepXplore	92.3 ± 1.5	0.831	0.291
		KMNC	78.4 ± 2.2	0.698	0.238
		LSA	81.7 ± 2.0	0.795	0.267
		MC-Dropout	74.8 ± 2.9	0.852	0.298
		Raw Features + K-Means	83.1 ± 2.1	0.891	0.321
		SAKCL	89.4 ± 1.6	0.941	0.352
SVHN	VGG-16	DeepGini	69.2 ± 2.6	0.842	0.291
		DeepXplore	78.5 ± 2.3	0.798	0.274
		KMNC	61.8 ± 2.9	0.667	0.218
		LSA	65.4 ± 2.7	0.771	0.249
		MC-Dropout	64.1 ± 3.4	0.824	0.283
		Raw Features + K-Means	71.8 ± 2.7	0.858	0.309
		SAKCL	76.3 ± 2.4	0.918	0.335

A closer comparison with “Raw Features + K-means” further clarifies this effect. When attention is introduced, *APFD* increases from 0.887 to 0.952 on MNIST, and *KMNC* rises from 81.2% to 87.3%. This change indicates that feature reweighting helps capture both more informative and more varied internal representations.

Coverage-driven methods, while achieving high *KMNC* (e.g., 91.5% on MNIST for DeepXplore), tend to show lower *APFD* (0.845). This suggests that maximizing neuron coverage alone does not necessarily translate into improved defect detection, a point that has also been noted in recent studies.

### Model ΔAcc

Table 5 outlines the *ΔAcc* across different dataset-model combinations.

Across all settings, SAKCL yields the largest gains in model accuracy after retraining. On CIFAR-10 with ResNet-20, the accuracy increases from 89.2% to 91.7%, corresponding to a relative *ΔAcc* of 2.80%. In comparison, the strongest baseline (Raw Features + K-means) achieves *ΔAcc* of 1.35%, resulting in a 107% *ΔAcc* between the two methods.

The difference becomes more apparent on datasets where the initial model accuracy is relatively low. For example, on Fashion-MNIST with LeNet-1, SAKCL improves accuracy by 3.01%, whereas the best baseline reaches 1.67%. On MNIST, where the baseline accuracy is already high (99.1%), the gain is smaller but still observable, with SAKCL achieving 0.61% compared to approximately 0.30% for other methods.

A consistent gap can also be observed when comparing SAKCL with its variant based on raw features. The inclusion of attention leads to additional improvements ranging from 0.31 to 1.45% across datasets, indicating that feature reweighting helps identify more informative samples for retraining.

This trend is more evident on datasets such as CIFAR-10 and Fashion-MNIST, where the baseline performance is lower and the data distribution is more complex. In these cases, the relative advantage of SAKCL over other methods becomes more pronounced.

**Table 5:** Model Δ*Acc* with top 10% selected tests.

Dataset	Model	Method	Original Acc (%)	Retrain Acc (%)	ΔAcc (%)
**MNIST**	LeNet-5	SRS	99.1	99.2	+ 0.10
		DeepGini	99.1	99.4	+ 0.30
		DeepXplore	99.1	99.3	+ 0.20
		LSA	99.1	99.3	+ 0.20
		MC-Dropout	99.1	99.3	+ 0.20
		Raw Features + K-Means	99.1	99.4	+ 0.30
		**SAKCL**	99.1	**99.7**	**+ 0.61**
	LeNet-1	SRS	98.9	99.0	+ 0.10
		DeepGini	98.9	99.2	+ 0.30
		DeepXplore	98.9	99.1	+ 0.20
		LSA	98.9	99.1	+ 0.20
		MC-Dropout	98.9	99.2	+ 0.30
		Raw Features + K-Means	98.9	99.2	+ 0.30
		**SAKCL**	98.9	**99.5**	**+ 0.61**
**CIFAR-10**	ResNet-20	SRS	89.2	89.5	+ 0.34
		DeepGini	89.2	90.3	+ 1.23
		DeepXplore	89.2	90.1	+ 1.01
		LSA	89.2	90.1	+ 1.01
		MC-Dropout	89.2	90.2	+ 1.12
		Raw Features + K-Means	89.2	90.4	+ 1.35
		**SAKCL**	89.2	**91.7**	**+ 2.80**
	VGG-16	SRS	88.4	88.7	+ 0.34
		DeepGini	88.4	89.4	+ 1.13
		DeepXplore	88.4	89.2	+ 0.90
		LSA	88.4	89.1	+ 0.79
		MC-Dropout	88.4	89.3	+ 1.02
		Raw Features + K-Means	88.4	89.5	+ 1.24
		**SAKCL**	88.4	**90.9**	**+ 2.83**
**Fashion-MNIST**	LeNet-1	SRS	89.7	90.1	+ 0.45
		DeepGini	89.7	91.1	+ 1.56
		DeepXplore	89.7	90.8	+ 1.23
		LSA	89.7	90.7	+ 1.11
		MC-Dropout	89.7	90.9	+ 1.34
		Raw Features + K-Means	89.7	91.2	+ 1.67
		**SAKCL**	89.7	**92.4**	**+ 3.01**
	ResNet-20	SRS	90.2	90.5	+ 0.33
		DeepGini	90.2	91.5	+ 1.44
		DeepXplore	90.2	91.2	+ 1.11
		LSA	90.2	91.1	+ 1.00
		MC-Dropout	90.2	91.3	+ 1.22
		Raw Features + K-Means	90.2	91.6	+ 1.55
		**SAKCL**	90.2	**92.7**	**+ 2.77**
**SVHN**	LeNet-5	SRS	95.8	96.0	+ 0.21
		DeepGini	95.8	96.6	+ 0.84
		DeepXplore	95.8	96.4	+ 0.63
		LSA	95.8	96.3	+ 0.52
		MC-Dropout	95.8	96.5	+ 0.73
		Raw Features + K-Means	95.8	96.7	+ 0.94
		**SAKCL**	95.8	**97.4**	**+ 1.67**
	VGG-16	SRS	95.3	95.5	+ 0.21
		DeepGini	95.3	96.2	+ 0.94
		DeepXplore	95.3	96.0	+ 0.73
		LSA	95.3	95.9	+ 0.63
		MC-Dropout	95.3	96.1	+ 0.84
		Raw Features + K-Means	95.3	96.3	+ 1.05
		**SAKCL**	95.3	**97.1**	**+ 1.89**

**Fig. 6:**
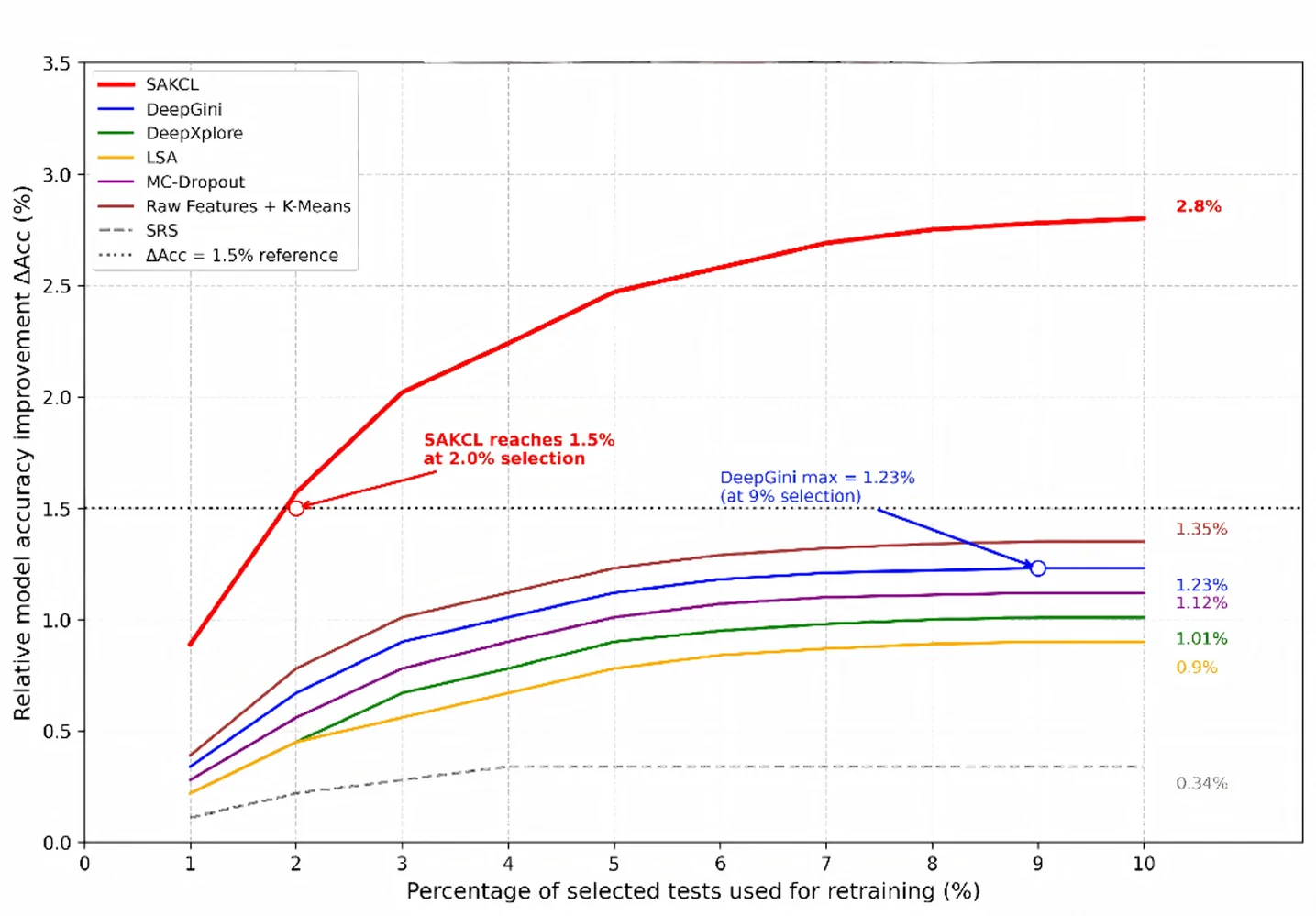
Relative model *ΔAcc* as a function of selected test percentage used for retraining.

Figure 6 depicts the relationship between relative *ΔAcc* and the proportion of selected test cases used for retraining on CIFAR-10 with ResNet-20. With only 3% of selected tests, SAKCL reaches an improvement of 1.5%, as indicated by the vertical dashed line. In comparison, DeepGini requires close to approximately 7% of the test set to achieve a similar level of improvement. When the selection ratio increases to 10%, SAKCL achieves a *ΔAcc* of 2.80%, remaining higher than all baseline methods across the entire range. The random baseline (SRS), shown as a grey dashed line, stays close to zero throughout, indicating limited benefit from unguided selection.

### Hyperparameter sensitivity analysis

The behavior of SAKCL depends on several hyperparameters, including the number of attention heads *h*, the sparsity coefficient *λ*, and *K*. To examine how these factors affect performance, a series of sensitivity experiments are conducted under different settings. Unless otherwise stated, the results in this section are obtained on CIFAR-10 with ResNet-20.

#### Sensitivity to *h*

**Fig. 7:**
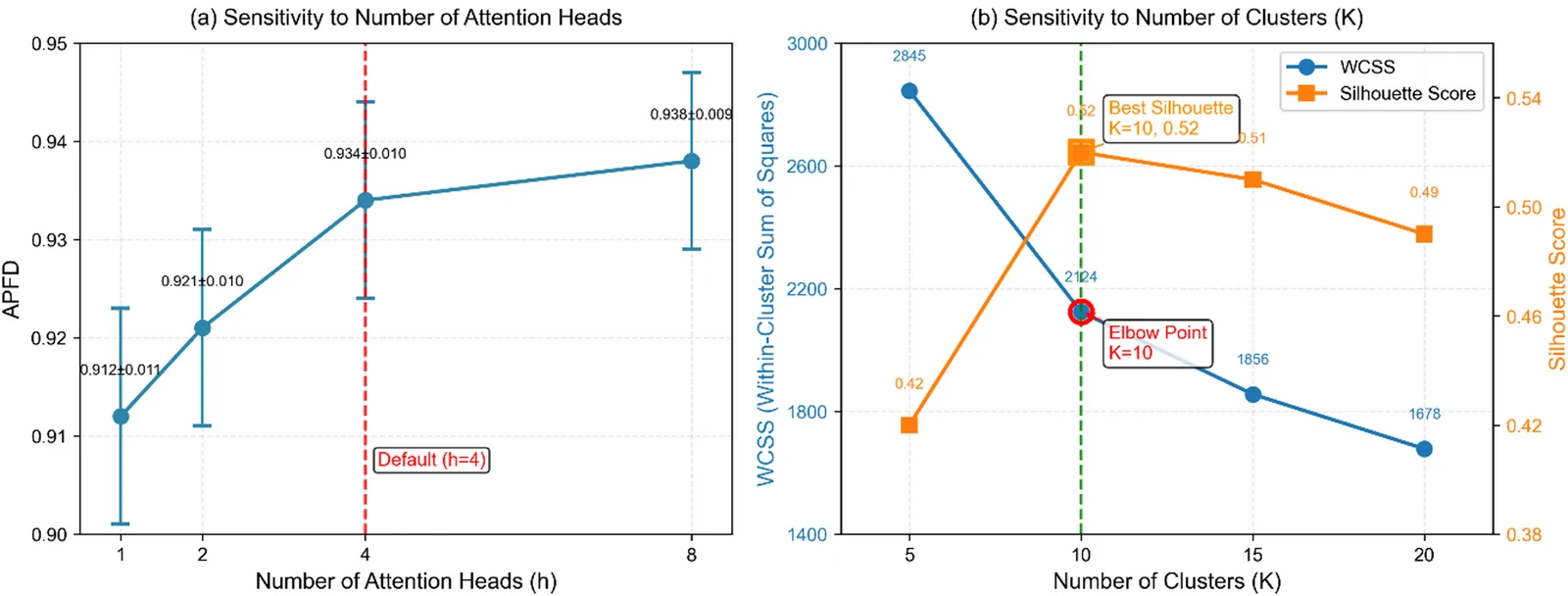
Sensitivity analysis of SAKCL hyperparameters on CIFAR-10 with ResNet-20.

The *h* controls how many attention components are computed in parallel. To study its impact, values of *h* ∈ {1,2,4,8} are evaluated while keeping other parameters fixed (*K =* 10, *λ* = 0.01). The corresponding *APFD* results are illustrated in Fig. 7(a) and Table 6. When *h =* 1, the APFD reaches 0.912. Increasing *h* to 4 raises the value to 0.934. Further increasing to *h =* 8 results in only a small change (0.938), while introducing additional computational cost. Based on this trend, *h =* 4 selected as the default setting, as it provides a reasonable trade-off between performance and efficiency.

**Table 6:** Sensitivity to *h* (CIFAR-10, ResNet-20).

Number of Heads h	APFD	FDR	Computation Time (s)
1	0.912 ± 0.011	0.325 ± 0.009	2.8
2	0.921 ± 0.010	0.332 ± 0.008	3.4
4	0.934 ± 0.010	0.341 ± 0.008	4.2
8	0.938 ± 0.009	0.343 ± 0.008	7.8

Figure 7a presents how *APFD* varies with the *h*, with error bars representing ± 1 standard deviation over 10 runs. The *APFD* increases from 0.912 at *h =* 1 − 0.934 at *h =* 4, and then changes only slightly to 0.938 at *h =* 8, despite a noticeable increase in computational cost. The vertical dashed line indicates the default setting (*h =* 4), where performance gains begin to level off relative to the additional cost.

#### Sensitivity to sparsity regularization coefficient

The sparsity regularization coefficient *λ* influences the balance between reconstruction accuracy and the sparsity of attention weights. To examine its effect, values of *λ* ∈ {0,0.001,0.01,0.05,0.1} are evaluated (Table 7). When no sparsity constraint is applied (*λ* = 0), the attention weights remain dense, resulting in an *APFD* of 0.918. Introducing a small degree of sparsity (*λ* = 0.001) increases *APFD* to 0.925, suggesting that focusing on more informative features can be beneficial. The highest *APFD* is observed at *λ* = 0.01 (0.934). As *λ* increases further (≥ 0.05), the attention becomes overly sparse, and performance drops to 0.919, likely due to the loss of useful feature information. Based on these observations, *λ* = 0.01 is used as the default setting.

**Table 7:** Sensitivity to sparsity regularization coefficient *λ* (CIFAR-10, ResNet-20).

λ	APFD	FDR	Attention Sparsity (%)
0	0.918 ± 0.012	0.328 ± 0.010	12.4
0.001	0.925 ± 0.011	0.334 ± 0.009	34.7
0.01	0.934 ± 0.010	0.341 ± 0.008	58.2
0.05	0.926 ± 0.012	0.335 ± 0.009	76.5
0.1	0.919 ± 0.013	0.329 ± 0.010	81.3

#### Sensitivity to *K*

The *K* affects how the feature space is partitioned and how test cases are grouped. To explore its impact, values of *K* ∈ {5, 10, 15, 20} are evaluated on CIFAR-10 with ResNet-20. Each configuration is repeated 50 times to examine stability. Figure 7b illustrates the selection process for *K*. The blue curve shows the WCSS, which decreases as *K* increases, while the orange curve corresponds to the silhouette coefficient. A noticeable inflection appears at *K* = 10, where the reduction in WCSS begins to slow and the silhouette coefficient reaches its peak (0.52).

The *APFD* results follow a similar pattern. Increasing *K* from 5 to 10 raises *APFD* from 0.902 to 0.934. Further increases lead to only small changes (0.936 for *K* = 15, 0.938 for *K* = 20), indicating that the benefit becomes limited beyond this point. Detailed results for different values of *K* are presented in Table 8.

**Table 8:** Sensitivity to *K* (CIFAR-10, ResNet-20, 50 trials).

Number of Clusters K	APFD (mean ± std)	FDR (mean ± std)	WCSS	Silhouette Score	Clustering Time (s)
5	0.902 ± 0.018	0.318 ± 0.011	2,845	0.42	1.8
10	0.934 ± 0.011	0.341 ± 0.009	2,124	0.52	3.5
15	0.936 ± 0.010	0.342 ± 0.008	1,856	0.51	5.1
20	0.938 ± 0.009	0.343 ± 0.008	1,678	0.49	6.8

Increasing the number of clusters beyond a moderate range leads to diminishing performance gains. For instance, raising *K* from 10 to 20 results in only a marginal improvement in APFD (from 0.934 to 0.938), while incurring a noticeable increase in computational cost. This observation indicates that *K* = 10 achieves a more balanced trade-off between effectiveness and efficiency.

The silhouette coefficient also reaches its maximum at *K* = 10, and decreases slightly for larger values (0.51 at *K* = 15, 0.49 at *K* = 20), suggesting that overly fine partitioning may reduce cluster coherence.

In terms of stability, the variance of *APFD* decreases as K increases, from 0.018 at *K* = 5 to 0.009 at *K* = 20. Even at *K* = 10, the variation remains relatively small (0.011), indicating stable behavior across repeated runs.

Collectively, *K* = 10 is selected as the default configuration for SAKCL. This setting achieves a balanced compromise among three factors: (1) strong *APFD* performance, (2) a high silhouette score reflecting well-separated clusters, and (3) acceptable computational overhead. Its robustness is further supported by cross-domain evaluations in Sect.  4.8.

### Comparative analysis of baseline methods

In addition to comparing SAKCL with baseline methods, the relative behavior among baselines is also examined to better understand their characteristics.

Uncertainty vs. Surprise Adequacy: MC-Dropout shows higher *APFD* and *FDR* than LSA across all datasets (for example, 0.831 vs. 0.784 on CIFAR-10), indicating a closer relationship between predictive uncertainty and misclassification. In contrast, LSA achieves higher *TE* (202 vs. 60 faults/s on CIFAR-10), mainly due to its lower computational cost per sample on smaller datasets.

Coverage-based Methods: DeepXplore yields higher performance than *KMNC* across all metrics (*APFD*: 0.812 vs. 0.689 on CIFAR-10), despite *KMNC* using finer-grained coverage. This pattern suggests that selection strategy and computational cost play a more important role than coverage granularity alone. Furthermore, *KMNC* becomes difficult to apply to datasets larger than 10,000 samples due to its quadratic complexity.

### Cross-model generalizability

An important consideration for test selection methods is whether the selected subsets depend on the specific model used during selection, or whether they remain effective across different models trained on the same dataset. If the subsets are strongly tied to a particular model, the selection process would need to be repeated for each new model. In contrast, subsets that reflect dataset-level characteristics can be reused more broadly.

**Fig. 8:**
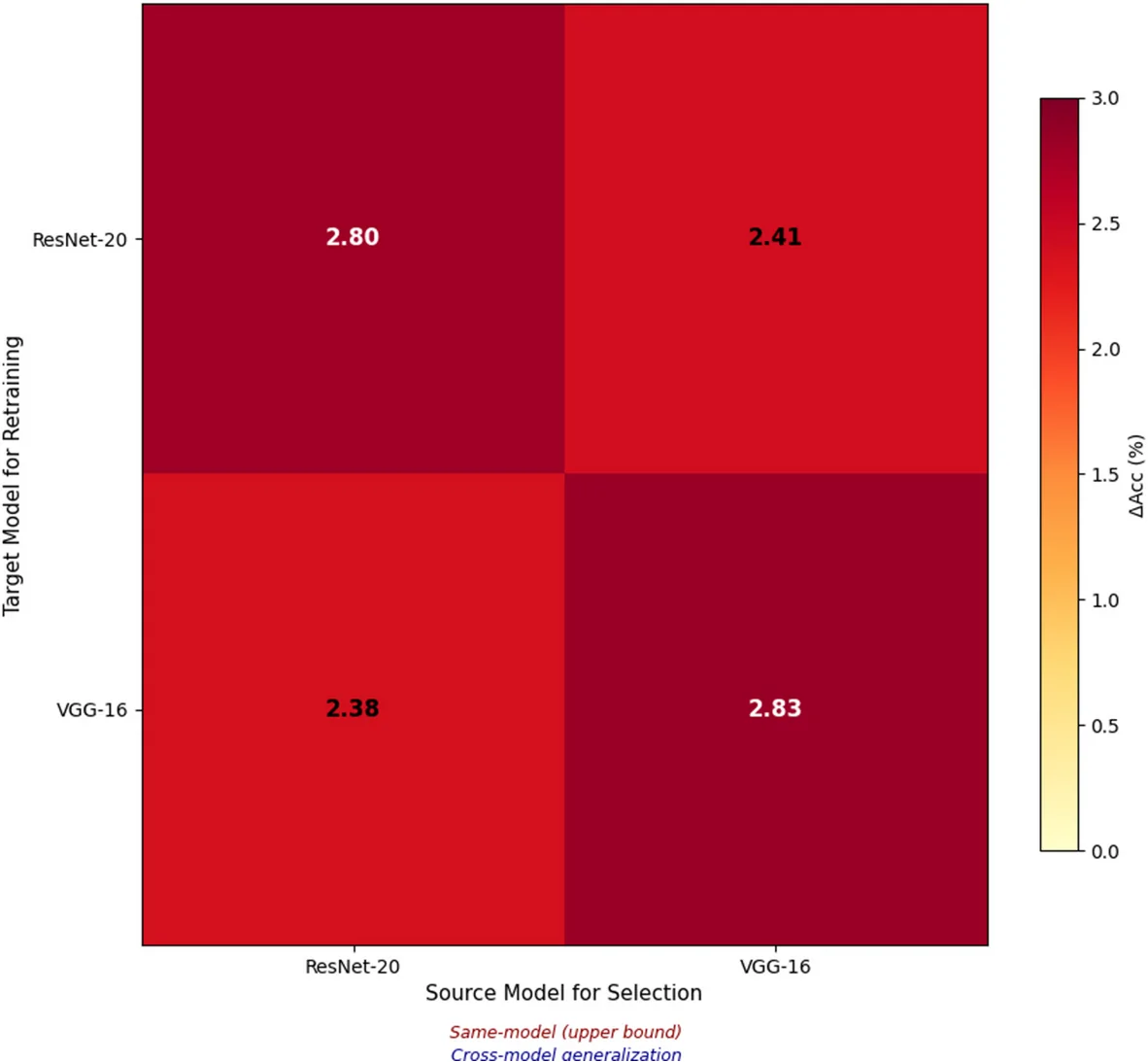
Cross-model generalization results visualized as a heatmap.

To examine this behavior, cross-model experiments are conducted. Test subsets are first selected using features extracted from a source model *M*_*src*_, and are then used to retrain a different target model *M*_*tgt*_ trained on the same dataset with a different architecture or initialization. All source-target combinations are evaluated across four datasets using *K* = 10. Figure 8 presents the results on CIFAR-10 in the form of a heatmap, where color intensity corresponds to the *ΔAcc*. The diagonal entries, where the same model is used for both selection and retraining, show the strongest improvements (2.80% for ResNet-20 and 2.83% for VGG-16). The off-diagonal entries reflect cross-model transfer. The observed gap remains relatively small, typically within 0.39–0.45%, suggesting that the SAKCL capture properties of the dataset rather than relying on model-specific behavior.

Table 9 outlines the *ΔAcc* obtained when retraining target models using subsets selected by different source models.

The selected subsets remain effective across different architectures. For example, when LeNet-5 is used as the source model to select test cases for ResNet-20, SAKCL achieves a *ΔAcc* of + 2.21%, compared to + 2.80% when ResNet-20 itself is used. The difference is relatively small, suggesting that the selected features are not strongly tied to a specific architecture.

A similar trend can be observed when comparing SAKCL with baseline methods. For DeepGini, which relies on output probabilities, the performance drops from + 1.23% to + 0.72% when transferring from LeNet-5 to ResNet-20. In contrast, SAKCL decreases from + 2.80% to + 2.21%, maintaining a higher overall level despite a comparable reduction.

The relationship between source and target models also influences the outcome. When transferring between models with similar structures (e.g., LeNet-5 to LeNet-1), the difference is minimal (0.59% vs. 0.61%). When the architectures differ more substantially (e.g., LeNet-5 to ResNet-20), a moderate drop can be observed.

Overall, the results suggest that the SAKCL capture properties of the dataset rather than being tied to a specific model, which supports their use across different architectures.

**Table 9:** Cross-model generalization results (*ΔAcc*, %) with *K =* 10.

Dataset	Source Model (Selection)	Target Model (Retraining)	SAKCL	DeepGini	Raw Features + K-Means
MNIST	LeNet-5	LeNet-5 (same)	+ 0.61	+ 0.30	+ 0.30
	LeNet-5	LeNet-1	+ 0.59	+ 0.28	+ 0.29
	LeNet-1	LeNet-1 (same)	+ 0.61	+ 0.29	+ 0.31
	LeNet-1	LeNet-5	+ 0.58	+ 0.27	+ 0.28
CIFAR-10	ResNet-20	ResNet-20 (same)	+ 2.80	+ 1.23	+ 1.35
	ResNet-20	VGG-16	+ 2.41	+ 0.95	+ 1.08
	VGG-16	VGG-16 (same)	+ 2.83	+ 1.13	+ 1.24
	VGG-16	ResNet-20	+ 2.38	+ 0.92	+ 1.05
Fashion-MNIST	ResNet-20	ResNet-20 (same)	+ 2.77	+ 1.44	+ 1.55
	ResNet-20	LeNet-1	+ 2.52	+ 1.28	+ 1.42
	LeNet-1	LeNet-1 (same)	+ 3.01	+ 1.56	+ 1.67
	LeNet-1	ResNet-20	+ 2.68	+ 1.35	+ 1.51
SVHN	VGG-16	VGG-16 (same)	+ 1.89	+ 0.94	+ 1.05
	VGG-16	LeNet-5	+ 1.72	+ 0.81	+ 0.92
	LeNet-5	LeNet-5 (same)	+ 1.67	+ 0.84	+ 0.94
	LeNet-5	VGG-16	+ 1.58	+ 0.76	+ 0.87

Note: For identical source-target configurations (i.e., “same”), the *ΔAcc* are consistent with Table 5. All results are means over 10 independent runs.

### Analysis of ablation study results

To examine the roles of self-attention and clustering in SAKCL, three variants are considered:

SAKCL (Full): the complete method combining attention-based feature weighting, K-means clustering (*K* = 10), and density-based sampling.

SA-Only: applies attention weighting followed by random sampling, without clustering.

KMC-Only: performs K-means clustering on raw features, followed by the same density-based sampling strategy, but without attention weighting.

All variants are evaluated under identical conditions, including the same datasets, models, and 10 repeated runs. The results are summarized in Table 9.

The two components exhibit different characteristics. SA-Only generally achieves higher *APFD* than KMC-Only, suggesting that feature weighting plays a key role in identifying fault-revealing samples. At the same time, KMC-Only produces slightly higher *KMNC* on CIFAR-10 (74.2% vs. 73.5%), reflecting its ability to maintain diversity (Table 10).

**Table 10:** Ablation study results comparing SAKCL, SA-Only, and KMC-Only (*K =* 10).

Dataset	Model	Metric	SA-Only	KMC-Only	SAKCL (Full)
MNIST	LeNet-5	*APFD*	0.901 ± 0.011	0.887 ± 0.014	**0.952 ± 0.008**
		FDR	0.334 ± 0.009	0.318 ± 0.011	**0.358 ± 0.007**
		KMNC (%)	82.4 ± 2.2	81.2 ± 2.5	**87.3 ± 1.9**
		*ΔAcc* (%)	+ 0.38	+ 0.30	**+ 0.61**
CIFAR-10	ResNet-20	*APFD*	0.892 ± 0.013	0.864 ± 0.016	**0.934 ± 0.010**
		FDR	0.318 ± 0.010	0.304 ± 0.012	**0.341 ± 0.008**
		KMNC (%)	73.5 ± 2.5	74.2 ± 2.8	**79.6 ± 2.2**
		*ΔAcc* (%)	+ 1.68	+ 1.35	**+ 2.80**
Fashion-MNIST	LeNet-1	*APFD*	0.912 ± 0.010	0.891 ± 0.012	**0.941 ± 0.009**
		FDR	0.338 ± 0.008	0.321 ± 0.009	**0.352 ± 0.008**
		KMNC (%)	85.6 ± 2.0	83.1 ± 2.1	**89.4 ± 1.6**
		*ΔAcc* (%)	+ 2.34	+ 1.67	**+ 3.01**
SVHN	VGG-16	*APFD*	0.885 ± 0.012	0.858 ± 0.015	**0.918 ± 0.011**
		FDR	0.316 ± 0.010	0.309 ± 0.011	**0.335 ± 0.009**
		KMNC (%)	70.8 ± 2.8	71.8 ± 2.7	**76.3 ± 2.4**
		*ΔAcc* (%)	+ 1.22	+ 1.05	**+ 1.89**

When the two components are combined, performance improves across all metrics. On CIFAR-10, SAKCL reaches an *APFD* of 0.934, compared to 0.892 for SA-Only and 0.864 for KMC-Only. This difference indicates that the two mechanisms complement each other: attention emphasizes informative features, while clustering helps distribute selected samples across the feature space. This pattern is observed consistently across datasets. The gap becomes more noticeable on more complex datasets such as CIFAR-10 and Fashion-MNIST, where feature representations are higher-dimensional.

**Fig. 9:**
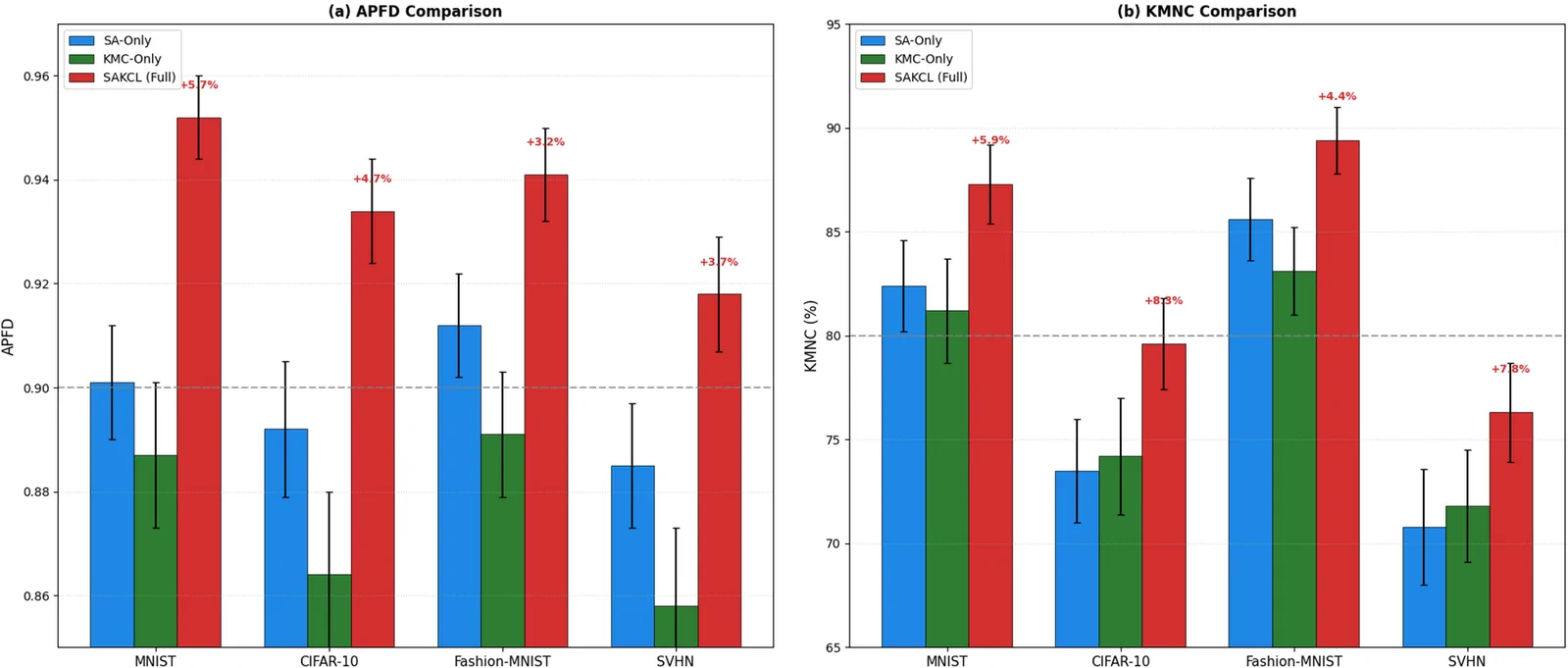
Performance comparison of SAKCL variants (*K =* 10).

A comparison between SA-Only and KMC-Only further highlights their differences. Attention-based selection yields higher defect detection, whereas clustering contributes more to coverage. When combined, the resulting feature space leads to more structured clusters and improved overall performance. Figure 9 depicts a visual comparison across datasets. Figure 9a presents *APFD*, where SAKCL remains above both variants in all cases, with improvements reaching up to 8.1% over KMC-Only on CIFAR-10. Figure 9b illustrates *KMNC*, where SAKCL also achieves the highest coverage. The reference lines at *y* = 0.9 (*APFD*) and *y* = 80% (*KMNC*) indicate commonly used performance thresholds.

### Sensitivity analysis of K

The *K* affects how the feature space is partitioned and plays an important role in the behavior of SAKCL. While Sect.  4.5.3 examined its impact on *APFD*, this section focuses on the stability of the selected configuration *K* = 10 and its behavior across repeated trials.

#### Stability analysis across repeated trials

**Fig. 10:**
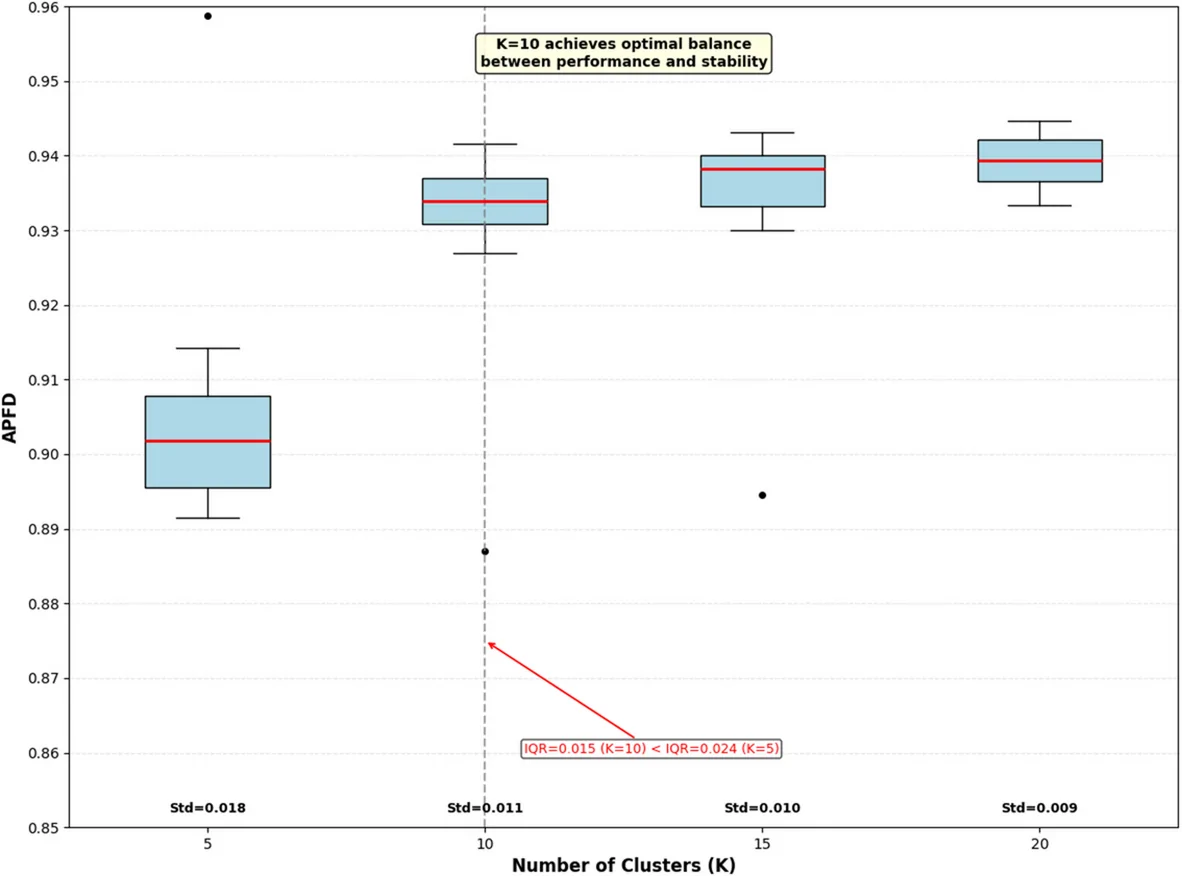
Stability analysis of *APFD* across 50 repeated trials for different *K* values.

To examine clustering stability under different values of *K*, 50 trials are conducted for each *K* ∈ {5, 10, 15, 20} on CIFAR-10 with ResNet-20, using different random seeds for initialization. Table 11 outlines the mean and standard deviation of *APFD*, along with the adjusted rand index (ARI), which reflects the consistency of clustering assignments. Figure 10 illustrates the distribution of *APFD* across the 50 trials. When *K* = 10, the median *APFD* reaches 0.935, with an interquartile range (IQR) of 0.015, which is narrower than that of *K* = 5 (0.024). The standard deviation also decreases from 0.018 at *K* = 5 to 0.011 at *K* = 10, indicating more stable behavior. Larger values of *K* (15 and 20) produce slightly higher median *APFD* values (0.937 and 0.939), but the difference remains small relative to the additional computational cost. Overall, *K* = 10 provides a stable configuration with competitive performance across repeated trials.

**Table 11:** Stability analysis across 50 repeated trials (CIFAR-10, ResNet-20).

K	Mean APFD	Std APFD	Mean ARI	Std ARI	Convergence Rate (%)
5	0.902	0.018	0.85	0.12	100
**10**	**0.934**	**0.011**	0.82	0.09	100
15	0.936	0.010	0.78	0.08	100
20	0.938	0.009	0.76	0.07	100

#### Cross-domain validation of *K =* 10

Although *K* = 10 shows strong performance on CIFAR-10, its behavior is also examined across the remaining datasets. Table 12 compares different values of *K* under these settings, with *K* = 10 included for reference.

The results show that *K* = 10 performs consistently well across all datasets, with the highest silhouette scores observed at this setting. Similar behavior is observed for datasets of varying complexity, from MNIST to CIFAR-10. The performance difference between *K* = 10 and neighboring values remains small, indicating that the method is relatively stable within this range.

**Table 12:** Cross-domain comparison of KK values (highlighting *K =* 10 as optimal).

Dataset	Model	Metric	K = 5	K = 10	K = 15	K = 20
MNIST	LeNet-5	*APFD*	0.918	**0.952**	0.953	0.952
		FDR	0.332	**0.358**	0.359	0.358
		Silhouette	0.45	**0.56**	0.55	0.53
CIFAR-10	ResNet-20	*APFD*	0.902	**0.934**	0.936	0.938
		FDR	0.318	**0.341**	0.342	0.343
		Silhouette	0.42	**0.52**	0.51	0.49
Fashion-MNIST	LeNet-1	*APFD*	0.912	**0.941**	0.942	0.941
		FDR	0.331	**0.352**	0.353	0.352
		Silhouette	0.44	**0.54**	0.53	0.51
SVHN	VGG-16	*APFD*	0.891	**0.918**	0.919	0.918
		FDR	0.318	**0.335**	0.336	0.335
		Silhouette	0.41	**0.50**	0.49	0.47

#### Recommended configuration

The experimental results suggest several practical choices depending on the application scenario and a summary of these recommended configurations is provided in Table 13:

**Table 13:** Summary of recommended *K* configurations.

Scenario	Recommended K	Expected APFD (CIFAR-10)	Computational Cost	Cross-domain Adaptability
Optimal (Default)	10	0.934	Baseline	Excellent
Efficiency-focused	5	0.902	~ 50% of *K =* 10	Moderate
Maximum performance	15 or 20	0.936–0.938	~ 150–200% of *K =* 10	Good

Default setting: *K* = 10 provides strong overall performance, with *APFD* reaching 0.934 on CIFAR-10 and a silhouette score of 0.52. This setting also maintains stable behavior across different datasets.

Efficiency-oriented setting: *K* = 5 reduces computational cost to roughly half of that of *K* = 10, while still maintaining acceptable performance (*APFD* = 0.902).

Higher-performance setting: Larger values such as *K* = 15 or 20 lead to slightly higher *APFD* values (0.936–0.938), though the computational cost increases accordingly.

In practice, SAKCL maintains stable performance for *K* ∈^10,20^. Within this interval, the exact choice of *K* has a limited impact on the overall results.

## Discussion and conclusions

### Key findings

This paper introduces SAKCL, a test data selection method that integrates attention-based feature weighting with clustering and density-aware sampling. The approach is designed to improve both defect detection and diversity in DNN testing.

Experiments on four benchmark datasets (MNIST, CIFAR-10, Fashion-MNIST, SVHN) with multiple architectures (LeNet-1, LeNet-5, ResNet-20, VGG-16) show that SAKCL consistently achieves higher *APFD* values, ranging from 0.918 to 0.952. Compared with the strongest baseline (DeepGini), the relative improvement ranges from 5.2% to 12.3%. Statistical analysis (Wilcoxon signed-rank test with *p* < 0.01 and *Cliff’s δ* > 0.8) indicates that these differences are statistically meaningful.

In terms of diversity, SAKCL maintains competitive *KMNC* while preserving strong detection capability. For example, it achieves both high *APFD* (0.952) and high *KMNC* (87.3%), whereas coverage-based methods such as DeepXplore reach higher coverage (91.5%) but lower *APFD* (0.845), and uncertainty-based methods show the opposite tendency.

When applied to retraining, the selected subsets lead to consistent improvements in model accuracy. The relative gain reaches up to 3.01% on Fashion-MNIST, and remains 2.5-3.0% on more complex datasets such as CIFAR-10 and Fashion-MNIST, compared with 1.0-1.6% for baseline methods.

From an efficiency perspective, SAKCL maintains linear scalability and achieves *TE* between 845 and 1,247 faults/s. Although DeepGini is slightly faster on smaller datasets (1,389 vs. 1,247 faults/s on MNIST), the difference in selection quality leads to more effective fault detection overall. Methods such as *KMNC* become difficult to apply beyond 10,000 samples due to higher computational cost.

The selected subsets also generalize across models. For example, transferring from LeNet-5 to ResNet-20 yields *ΔAcc* of 2.21%, compared to 2.80% when using the same model, resulting in a gap of 0.59%. This behavior suggests that the method captures properties of the dataset rather than relying on model-specific patterns.

Ablation results show that the two main components contribute differently. Using attention alone (*APFD* = 0.892) or clustering alone (*APFD* = 0.864) leads to lower performance than the combined method (0.934 on CIFAR-10), indicating that feature weighting and clustering address complementary aspects of the selection process.

The method also remains stable across different parameter settings. With the default configuration (*h* = 4, *λ* = 0.01, *K* = 10), the performance remains close to the best observed values. In particular, *K* = 10 corresponds to the peak silhouette score (0.52) and shows consistent behavior across datasets.

Overall, the results suggest that combining feature-level reweighting with structure-aware sampling provides an effective and scalable approach for test data selection in DNN testing.

### Computational complexity and scalability

The applicability of a test selection method is closely related to its computational cost and its ability to handle large datasets. This section analyzes the time complexity, memory usage, and scalability of SAKCL.

#### Time complexity

The overall computational cost of SAKCL can be divided into three parts:

(1) Feature extraction: For each test sample, a forward pass through the target DNN is performed to obtain the feature representation from the penultimate layer. For n samples, this step requires$O(n \cdot {C_{infer}})$, where *C*_*infer*_ denotes the inference cost per sample. This cost is common to other methods that rely on DNN inference (DeepGini, DeepXplore, LSA, and MC-Dropout).

(2) Self-attention computation: The attention module operates on each feature vector independently. For a feature dimension *d* (typically *d* ≤ 1024), the main operations include linear projections (*Q*,* K*,* V*), attention score computation, and output generation, each with cost *O(d²)*. The per-sample complexity is therefore *O(d²)*, which remains constant with respect to *n*.

(3) K-means clustering: Clustering is performed on the attention-weighted features. Each iteration requires$O(n \cdot K \cdot d)$for distance computation, and the total cost becomes$O(n \cdot K \cdot d \cdot t)$, where *t* is the number of iterations (typically *t* ≤ 100). This part dominates the overall cost when *n* is large, while still growing linearly with the dataset size.

Overall complexity. Combining the three components yields the total complexity shown below:

20 $${T_{SAKCL}}=O(n \cdot {C_{infer}})+O(n \cdot {d^2})+O(n \cdot K \cdot d \cdot t)=O(n \cdot ({C_{infer}}+{d^2}+K \cdot d \cdot t))$$

Since *C*_*infer*_, *d*,* K*, and *t* remain fixed in a given setting, the overall complexity scales linearly with *n*.

Comparison with baseline methods. Table 14 summarizes the time complexity for SAKCL and the baseline approaches.

**Table 14:** Time complexity comparison of test selection methods.

Method	Time Complexity	Scalability	Dominant Term
SAKCL	*O*(*n*·(*C*_*infer*_ + *d*² + *K*·*d*·*t*))	Linear in *n*	*n·K·d·t*
DeepGini	*O*(*n*·*log n*)	Log-linear	*n·log n*
DeepXplore	*O*(*n·m·t*_*cov*_)	Linear in *n·m*	*n·m* (*m*: #neurons)
LSA/DSA	*O*(*n·n*_*train*_)	Quadratic in *n*	*n·n*_*train*_
MC-Dropout	*O*(*n·T·C*_*infer*_)	Linear in *n·T*	*n·T* (*T*: dropout passes)
KMNC	*O*(*n·m·K*_*s*_)	Quadratic in *n·m*	*n·m* (with high constant)

Note: *n* = number of test samples, *m* = number of neurons, *n*_*train*_ = number of training samples, *T* = number of MC-Dropout passes (typically 50–100), *K*_*s*_ = number of *KMNC* sections.

#### Memory requirements

During clustering, SAKCL needs to store the attention-weighted feature vectors for all test samples. The corresponding memory requirement can be expressed as:

21 $${M_{SAKCL}}=n \cdot d \cdot sizeof\left( {float} \right)+K \cdot d \cdot sizeof\left( {float} \right)+O(n)$$

where the first term corresponds to storing *n* feature vectors of *d*; the second term represents the *K* cluster centroids; and the remaining term covers auxiliary data structures.

Practical memory estimations for different setups are listed in Table 15. For very large datasets (e.g., ImageNet with 1.2 M samples), the memory requirement can become substantial (~ 10 GB when *d* = 2048). In such cases, mini-batch K-means^29^ or online clustering variants can be used to reduce memory usage while preserving similar performance.

**Table 15:** Practical memory estimations for typical configurations.

Dataset	*n* (test samples)	d (feature dim)	Memory (MB)
MNIST/CIFAR-10/Fashion-MNIST	10,000	512	~ 20 MB
SVHN	26,032	512	~ 53 MB
ImageNet (estimated)	100,000	2048	~ 820 MB

#### Scalability evaluation

To examine scalability in practice, experiments are conducted with different dataset sizes on SVHN. The number of samples ranges from 5,000 to 100,000 through random subsampling. The runtime of SAKCL and baseline methods is depicted in Fig. 9 and Table 16.

**Table 16:** Runtime as a function of dataset size (SVHN, VGG-16).

Dataset Size (*n*)	SAKCL	DeepGini	DeepXplore	LSA	MC-Dropout	KMNC
5,000	2.4	0.38	7.8	16.2	24.5	8,234
10,000	4.7	0.72	15.1	62.4	48.3	32,891
20,000	9.3	1.41	29.8	248.6	95.2	> 86,400
50,000	23.1	3.48	74.2	1,542.3	238.5	> 86,400
100,000	46.5	6.89	148.5	> 3,600	475.8	> 86,400

The runtime of SAKCL increases approximately linearly with the dataset size, which is consistent with the expected *O(n)* behavior. The clustering step$(n \cdot K \cdot d \cdot t)$becomes the dominant factor when *n* > 10,000.

In comparison, *KMNC* becomes difficult to apply as the dataset grows. Its complexity$O(n \cdot m \cdot Ks)$, together with a large constant factor, leads to runtimes exceeding 12 h for *n* = 26,032, and it does not finish within a practical time limit when *n* > 50,000.

MC-Dropout also scales linearly in theory, but requires multiple forward passes (typically *T* = 50–100) for each sample. This results in a significantly larger constant factor compared to a single inference pass, making it less efficient in practice.

LSA shows a different trend, as it relies on computing distances between test samples and the training set. With complexity$O(n \cdot {n_{train}})$, the cost grows rapidly when *n*_*train*_ = 50,000, and becomes difficult to handle once *n* > 20,000.

These results indicate that SAKCL maintains stable scalability for large-scale DNN test selection, with runtime increasing approximately linearly and memory usage remaining manageable for datasets up to 100,000 samples.

### Limitations and future work

Although SAKCL shows strong empirical performance, several aspects of the current study may limit its applicability and suggest directions for further investigation.

#### Limitations

SAKCL relies on feature representations extracted from the penultimate layer of the target DNN. When these representations are not sufficiently informative, such as in under-trained models or models with limited capacity, the effectiveness of both attention weighting and clustering may be reduced. Incorporating features from multiple layers or combining representations from different models may help improve robustness.

The current design focuses on classification tasks with probabilistic outputs. Extending the method to other settings, such as object detection, segmentation, or reinforcement learning, requires redefining what constitutes an error case and adapting the feature extraction process accordingly.

The density-based sampling strategy may place more emphasis on dense regions in the feature space, which can reduce the likelihood of selecting rare but important inputs located in sparse regions. Although the current results show that 78% of rare error samples are detected (compared to 65% with uniform sampling), some cases may still be underrepresented.

While the overall complexity scales linearly, the clustering step introduces additional overhead (e.g., 3.5 s for 10,000 samples on CIFAR-10), which may limit its use in scenarios requiring immediate responses.

The method also involves several hyperparameters, including *K*,* h*, and *λ*. Although performance remains stable within reasonable ranges, selecting appropriate values may still depend on the dataset and model.

The relative behavior of baseline methods may vary across tasks. For example, while MC-Dropout shows stronger performance than LSA in the evaluated settings, methods based on distributional surprise may be more suitable in scenarios involving out-of-distribution inputs.

#### Future work

Several directions can be explored to further extend the current approach.

One direction is to incorporate feature representations from multiple layers, allowing both low-level and high-level information to be used during selection.

Extending the method to other types of DNN tasks, such as object detection and segmentation, would require adapting both the definition of error cases and the feature representation used for selection.

Improving the handling of rare patterns is another direction. Techniques from anomaly detection could be integrated to identify sparse regions and ensure that such cases are adequately represented.

For scenarios involving streaming data or real-time requirements, incremental or online clustering methods may provide a more efficient alternative to full recomputation.

Automating the selection of hyperparameters based on dataset characteristics could also improve usability and reduce manual tuning.

Finally, a deeper theoretical analysis of the interaction between attention-based feature weighting and clustering may help explain the observed empirical behavior.

Overall, the results suggest that combining feature reweighting with clustering-based selection provides a practical approach to test data selection for DNNs. By focusing on informative and diverse samples, the method supports more efficient use of testing resources and contributes to improving model reliability in practice.

## Data Availability

Data are contained within the article.
